# A Pneumatic Flexible Linear Actuator Inspired by Snake Swallowing

**DOI:** 10.1002/advs.202405051

**Published:** 2024-09-12

**Authors:** Yuyan Qi, Jiaqi Shao, Yongjian Zhao, Tong Niu, Yi Yang, Songyi Zhong, Shaorong Xie, Yangqiao Lin, Yang Yang

**Affiliations:** ^1^ School of Mechatronic Engineering and Automation Shanghai University Shanghai 200444 China; ^2^ School of Computer Engineering and Science Shanghai University Shanghai 200444 China

**Keywords:** biomimetic design, flexible linear actuator, pneumatic actuation, versatility

## Abstract

Soft robots spark a revolution in human–machine interaction. However, developing high‐performance soft actuators remains challenging due to trade‐offs among output force, driving distance, control precision, safety, and compliance. Here, addressing the lack of long‐distance, high‐precision flexible linear actuators, an innovative pneumatic flexible linear actuator (PFLA) is introduced, inspired by the smooth and controlled process observed in snakes ingesting sizable food, such as eggs. This PFLA combines a soft tube, emulating the snake's body cavity, with a pneumatically driven piston. Through the joint modulation of moving resistance and driving force by pneumatic pressure, the PFLA exhibits exceptional motion control capabilities, including self‐holding without pressure supply, smooth low‐speed motion (down to 0.004 m s^–1^), high‐speed motion (up to 5.6 m s^–1^) with low air pressure demand, and a self‐protection mechanism. Highlighting its adaptability and versatility, the PFLA finds applications in various settings, including a wearable assistive devices, a manipulator capable of precise path tracking and positioning, and rapid transportation in diverse environments for pipeline inspection and firefighting. This PFLA combines biomimetic principles with sophisticated fluidic actuation to achieve long‐distance, flexible, precise, and safe actuation, offering a more adaptive solution for force/motion transmission, particularly in challenging environments.

## Graphical abstract

Soft robots revolutionize human–machine interaction, yet high‐performance actuators face challenges balancing force, distance, precision, and safety. This study introduces a pneumatic flexible linear actuator (PFLA) inspired by snake locomotion, achieving precise, low‐ and high‐speed movement with low pressure. It excels in wearable devices, precise manipulators, and pipeline inspection, combining biomimetic principles with fluidic actuation for flexible, precise, and safe operation.

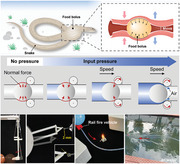

## 1 Introduction

Soft actuators are critical components of soft robots. Researchers have developed a series of soft actuators that introduce a spectrum of lightweight, efficient, and versatile motion paradigms, including bending,^[^ ^1^, ^2^ ^]^ twisting,^[^ ^3^, ^4^ ^]^ expansion,^[^ ^5^, ^6^ ^]^ contraction,^[^ ^7^, ^8^ ^]^ and elongation.^[^ ^9^, ^10^ ^]^ Among these, linear motion (encompassing sliding, contraction, elongation, etc.) is a fundamental movement paradigm employed in diverse applications, including wearable devices,^[^ ^11^, ^12^ ^]^ crawling robots,^[^ ^13^, ^14^ ^]^ exploration and rescue equipment,^[^ ^15^, ^16^ ^]^ and more. Three soft linear motion actuator types have already been developed: muscle‐type, growth‐type, and sliding‐type actuators.

Muscle‐type actuators mimic the action of biological muscles and generate reversible linear motion through material strain. Pneumatic artificial muscles (PAMs) have attracted widespread attention for their potential in economy, simplicity, and mechanical properties. McKibben PAMs, a typical type of muscle, can generate considerable contraction force, but their maximum contraction ratio is less than 40%.^[^ ^17^, ^18^, ^19^ ^]^ Vacuum‐driven PAMs offer a maximum contraction ratio of 90% while maintaining a high load capacity.^[^ ^20^, ^21^, ^22^ ^]^ However, their performance is limited by the maximum negative pressure difference (1 atm) they can provide. Recently, positive pressure‐driven PAM has achieved a higher contraction ratio of 92.9%.^[^ ^23^ ^]^ Although muscle‐type linear actuators achieve high expansion ratios, their linear motion is typically derived from other forms of movement. Positive pressure‐driven actuators generally achieve linear motion through radial expansion, while negative pressure‐driven actuators typically achieve it through the folding and contraction of the body, making it difficult for these actuators to achieve a full 100% contraction ratio. In contrast, growth‐type actuators, inspired by the tip growth observed in plants, achieve long‐distance movement through end elongation. Inflatable growth robots lengthen their bodies by everting an inverted membrane under pressure, achieving an extension ratio of more than 1000%.^[^ ^24^, ^25^ ^]^ Among these robots, one developed by Hawkes et al. demonstrates elongation of 72 m, with a maximum speed reaching 10 m s^–1^.^[^ ^26^ ^]^ Nevertheless, the critical bending and buckling loads of inflatable growth robots are often low, resulting in a limited payload capacity at the tip.^[^ ^27^, ^28^ ^]^ In contrast, growth robot systems based on additive manufacturing technology are more likely to support larger tip payloads, up to 100 N.^[^ ^29^, ^30^ ^]^ However, the extension speed of such robots tends to be quite slow, typically ranging from 1 to 10 mm min^–1^. While growth robots can extend with sufficient material supply, they are limited by unidirectional force transmission. The third variant mirrors the function of rigid lead screw‐slider mechanisms or track sliders, with a flexible tubular structure serving as a transmission path and a movable component for force/displacement output. This kind of structure provides significant load capacity and actuation distance, and can reversibly transmit force. Movable components can be categorized into active and passive types based on their actuation principles. For the active type, the input energy acts on the movable component and directly controls its motion, but this method often results in complex component designs and increased size and weight.^[^ ^31^, ^32^ ^]^ Movable components of the passive type move through the deformation of the flow channel under the input pressure, characterized by simple design, rapid response, and easy implementation. The flexible pneumatic cylinder can generate an output force of 16 N and move at a speed of approximately 1 m s^–1^ under a pressure of 500 kPa.^[^ ^33^ ^]^ In contrast, operating within the same pressure range, the hollow shaft actuator can generate a maximum force of 19 N and provide a wider speed range of 2–6.5 m s^–1^.^[^ ^34^ ^]^ However, the high speed poses challenges for precise positioning and speed control, with a 1 kPa change in air pressure leading to speed variations ranging from 21.7 to 46 mm s^–1^. This implies that even slight fluctuations in input pressure can lead to significant changes in speed. In addition, the reel actuator and the “λ‐actuator,”^[^ ^35^, ^36^ ^]^ which use a flat tube, achieve movement speeds below 1 m s^–1^ at an air pressure of less than 250 kPa. Nevertheless, the compliance of the flat tube is not as good as that of an elastic tube, making it prone to unwanted buckling during operation. Precisely controlling the motions of flexible linear robots has proven difficult without sacrificing compliance. Furthermore, due to the low stiffness of materials, they struggle to implement safety features commonly found in rigid transmission devices. For example, traditional rigid devices can achieve functions like self‐locking and backdrive prevention through design, but these features are challenging to realize in soft actuators due to their high flexibility and susceptibility to deformation. Currently, achieving wide speed modulation along with enhanced operational safety remains a significant challenge for flexible linear actuators. In nature, snakes can swallow food, such as eggs, larger than their own body diameter by adjusting the diameter of their body chamber through muscle contractions and relaxations.^[^ ^37^, ^38^ ^]^ Inspired by this process, we proposed the PFLA: an internal spherical piston set within a soft guide tube, an external slider is propelled through the piston's movement powered by pressurized air. Unlike previous designs, our approach exploits the soft tube's elasticity to adjust moving resistance, achieving a controllable balance between propulsion and friction forces. In the absence of driving pressure, the spherical piston, which is slightly larger than the tube diameter, is locked inside the tube. When pressure is applied, pneumatic pressure expands the tube diameter and initiates the piston's motion. At lower pressures, controlled expansion ensures stable, slow actuation; the lowest stable movement speed can go down to 0.004 m s^–1^ without encountering the “stick‐slip motion” phenomenon common in other pneumatic actuators (such as cylinders) at low speeds. At higher pressures, further expansion of the tube reduces moving friction, enabling fast movement at the low input pressures and enhancing efficiency in rapid movements; the highest stable movement speed can reach up to 5.6 m s^–1^ with an input pressure of 200 kPa. When the applied pressure continues to increase beyond a predetermined setting (usually when the input pressure is too high or overloaded), the tube's diameter continues to enlarge, allowing the pressurized airflow to overflow from the gap between the piston and the tube. At this stage, the driving force gradually stops increasing with input pressure, achieving the self‐protection function. The PFLA mimics the dynamic body size adjustment mechanism observed in snake swallowing, providing the actuator with a wide speed modulation range. This mechanism also incorporates two safety features: self‐holding and self‐protection. Comprehensive evaluations of the PFLA's speed characteristics, responsiveness to pneumatic fluctuations, and the reliability of its safety features have been conducted. Its extensive applicability in wearable technologies, rotary joint control, and applications in diverse environments showcases its capability as a flexible linear actuator for a wide range of devices. This pneumatic actuator offers a simple, powerful solution for driving flexible or rigid mechanical systems.

## 2 Results

### 2.1 Principle and Structure of the PFLA

**Figure 1:**
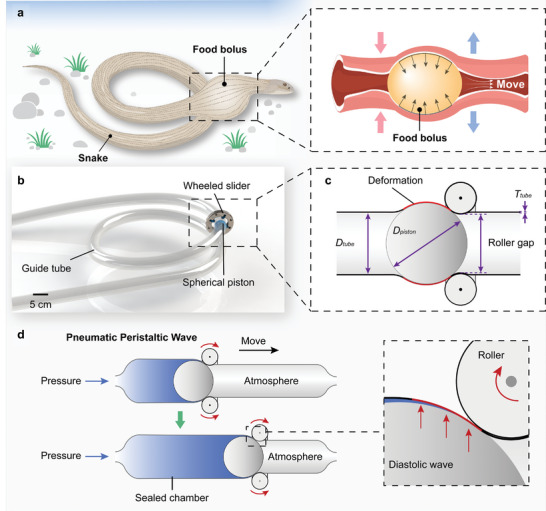
PFLA's composition and actuation principle. a) Schematic diagram of snake's body cavity peristalsis pushing food bolus. b) The PFLA inspired by snake swallowing comprises a soft guide tube, a slider, and a spherical piston. c) Key design parameters. d) Conceptual diagram illustrating the pneumatic peristaltic wave (PPW) utilized in the PFLA.

Snakes achieve the directional transportation of food bolus through the constraining and propelling action of muscle group wave peristalsis. They demonstrate remarkable swallowing ability due to their powerful adjustment of the body cavity (**Figure** **1**a). Drawing inspiration from this swallowing mechanism, we propose a PFLA based on pneumatic peristaltic wave (PPW, a diastolic wave formed in the guide tube wall under pneumatic pressure, used to drive the slider), utilizing the peristaltic propulsion mechanism in reverse. The PFLA consists of an elastic, soft guide tube, a spherical piston, and an annular wheeled slider (Figure 1b). The soft guide tube simulates the snake's body cavity and possesses elasticity and flexible deformation capabilities. The piston is inside the tube and experiences a constrained force from the tube wall. This constraint force can be adjusted by changing the tube diameter under the input pressure. The slider is sleeved on the tube, serving as a loading platform. Compared with snake propelling its internal objects through active chamber deformation, the PPW mechanism propels its external slider through passive tube deformation. The principle of PPW mechanism is illustrated in Figure 1d. As the piston moves along the tube under input pressure, it induces successive passive deformation of the tube wall, generating a forward‐propagating diastolic wave. This wave contacts the rollers on the slider, providing them with force and torque (Movie S1, Supporting Information).

In this design, the tube functions as a guide and provides a channel for the transmission of pneumatic energy. The piston blocks the airflow channel and causes deformation of the tube wall at its position. When the input pressure is sufficient, the piston can convert pneumatic energy into force and displacement to propel the slider forward. The slider and the piston collectively constitute a movable system capable of moving along the guide tube. The PFLA operates without the need for continuous inflation and deflation, resulting in low air consumption. To achieve PPW actuation, the diameter of the piston (*D*_*piston*_) must be larger than the inner diameter of the guide tube (*D*_*tube*_) to ensure the creation of a sealed chamber and the deformation of the tube wall (Figure 1c). Four rollers are evenly distributed along the circumference of the slider and gently pressed against the tube's surface. The roller gap of the slider' the distance between the edges of the rollers on the opposite sides' should be smaller than the sum of the piston diameter and twice the tube wall thickness (*T*_*tube*_). All components of the PFLA are made of widely available materials and manufactured using basic techniques, allowing its actuation distance and slider configuration to be customized as needed without introducing extra manufacturing challenges or costs. We fabricated a typical PFLA, which is characterized by a slider with an 8.5 mm roller gap, a piston with a diameter of 9 mm, and a guide tube measuring 8 mm (inner diameter) by 10 mm (outer diameter) (see Figure S4, Supporting Information).

### 2.2 PFLA's Performance Characterization

**Figure 2:**
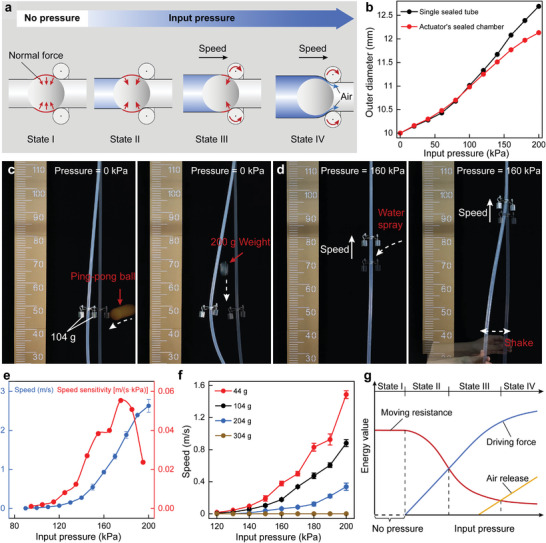
Performance characterization of the PFLA. a) The diameter of the sealed chamber changes with increasing input pressure. b) Comparison of the changes in diameters between a single sealed tube and the actuator's sealed chamber with air pressure. c) Speed and speed sensitivity of the typical PFLA under different input pressures. d) The speed of the movable system with loads of 44, 104, 204, and 304 g, under various input pressures. e) Diagram showing the changing of driving force, moving resistance, and air release with the increase in input pressure. f) Initial and final positions of the movable system with a 104 g load after being impacted by a 2.8 g ping‐pong ball and a 200 g weight without applying pressure. g) Operation of the movable system with a 104 g load under shaking and liquid spraying conditions. In (c) and (d), points are averages of three trials, and error bars show ± SD.

The diameter of the sealed chamber varies with the input pressure (Movie S2, Supporting Information), thereby regulating the moving resistance and performance of the PFLA. As shown in **Figure** **2**a, when there is no pressure input, the deformed guide tube exerts a large normal force on the piston surface, firmly anchoring it inside the airflow channel (state I). With the increase in input pressure, the sealed chamber expands, resulting in a decrease in the normal force applied to the piston surface (state II). When the movable system overcomes moving resistance and begins to move, the normal force acting on the piston will continue decreasing with the increase in input pressure (state III). The sealed chamber expands further with the increase in pressure, eventually forming a gap between the piston and the channel surface. This gap allows the sealed chamber to establish a connection with atmospheric pressure and slows down the expansion process by releasing air, thereby preventing the rupture of the guide tube (state IV). The diameter of a single sealed tube (black line) and a typical PFLA's sealed chamber (red line) with the increase in input pressure is compared (Figure 2b). The single sealed tube refers to a tube with one end connected to an air source and the other end sealed, made of the same material as the actuator's guide tube. The diameter of the single tube increases approximately linearly, while the rate of increase in the sealed chamber gradually decreases due to the formation of a gap. The regulation of the input pressure on the sealed chamber provides the PFLA with several distinctive advantages, including the self‐holding characteristic of the movable system, the wide speed modulation from micro to macro at low working pressure, and self‐protection. The stress distribution of the guide tube can be visualized through a COMSOL simulation (Figure S5b, Supporting Information), and the mathematical model for force analysis is provided in the Note S2 and Note S3 (Supporting Information).

We tested the self‐holding ability of a typical PFLA, which is defined as the minimum force required to induce reverse movement of the movable system without applying pressure. See Note S1 for (Supporting Information) all definitions. Due to the deformable property of soft materials, the self‐holding of the PFLA can be achieved under limited force. Using the experimental setup shown in Figure S10a (Supporting Information), we determined the upper limit of the self‐holding force to be 9.73 N, which is approximately 256 times the weight of the movable system. To verify the self‐holding capability of the PFLA, the slider (loaded with 104 g) was impacted by a ping‐pong ball and a 200 g weight, respectively, without input pressure. Results show that the PFLA remains firmly stable in its current position (Figure 2c). Moreover, during actuation, we sprayed water on the movable system, shook the guide tube, and observed that the movable system still ran stably, demonstrating its good anti‐interference ability (Figure 2d; Movie S3, Supporting Information). Furthermore, the mathematical model shows that, in addition to the anchoring effect of the deformed guide tube, the applied load amplifies the normal force, generating an additional force on the piston to enhance its anchoring effect (Figure S2, Supporting Information). This is similar to the self‐locking principle found in rigid screw transmission systems. We subsequently studied the movable system's speed response to the input pressure. The speed was measured by varying the input pressure from 0 to 200 kPa using the experimental setup shown in Figure S6 (Supporting Information). The movable system started moving at 90 kPa input pressure, with an average speed of 0.007 m s^–1^. (Figure 2e). The speed increased with the input pressure, reaching 2.63 m s^–1^ at 200 kPa. Additionally, using the experimental setups shown in Figure S17 (Supporting Information), we tested the repeatability of the PFLA's speed and its response time. The results indicate that the PFLA exhibits good repeatability and a rapid response, with detailed data provided in Figure S18 (Supporting Information). A comparison of the PFLA's speed under different input pressures is presented in Movie S4 (Supporting Information). The speed sensitivity is defined as the increment of speed corresponding to a 1 kPa increase in input pressure. As the input pressure increased, the overall trend of speed sensitivity initially rose and then decreased (Figure 2e). This increase can be attributed to the decrease in the normal force acting on the piston (state III), while the decrease results from the energy loss caused by air release exceeding the positive effect of friction reduction (state IV). In the following parts, we refer to the minimum input pressure required to move the movable system, the speed under the input pressure of 200 kPa, and the input pressure corresponding to the maximum speed sensitivity as the starting pressure, maximum speed, and inflection pressure, respectively. Air release from the sealed chamber was effectively visualized through finite element method (FEM) simulations in COMSOL (Movie S5, Supporting Information). We then measured the PFLA's speed under loads of 44, 104, 204, and 304 g. As the applied load increased, the speed of the movable system decreased (Figure 2f). Notably, with a load of 304 g, the movable system failed to move. The reason is that the sealed chamber undergoes significant expansion, creating a gap between the piston and the channel surface under a large load. This, in turn, results in the release of a substantial amount of air from the gap, thereby limiting the driving force acting on the piston. This behavior serves as a self‐protection mechanism under overload, preventing excessive strain on the elastic guide tube and prolonging its service life. It is analogous to the slip phenomenon observed in V‐belt drives. To assess the self‐protection performance under overload, we defined the load capacity as the maximum load that the movable system can effectively drive. The load capacity of the typical PFLA was quantified as 2.9 N (76 times the weight of the movable system) using the experimental setup shown in Figure S7 (Supporting Information). For a detailed explanation of the relationship between input pressure, load, and air release, refer to Figure S8 (Supporting Information). We explain the relationship between PFLA performance and input pressure from an energy perspective, as illustrated in Figure 2g. The proposed PFLA achieves self‐holding through the inherent elasticity of the guide tube and structural design, eliminating the need for external energy or supplementary mechanisms (state I). This feature contributes to enhancing safety and substantially reducing energy consumption. In the early stage of state III, the normal force on the piston remains significant, accompanied by a large moving resistance, resulting in the primary conversion of input pressure into friction loss. Consequently, the PFLA can execute precise operations and resist air pressure fluctuations to some extent. As the input pressure rises to the later stage of state III, both the normal force acting on the piston and the moving resistance are smaller. In this scenario, a higher proportion of pneumatic energy is used for driving, allowing the PFLA to operate at high speeds even with low air pressure and to respond sensitively to input changes. The PFLA achieves self‐protection under overload by releasing air as a result of the expansion of the sealed chamber (like state IV), without the need for load slip or additional equipment, thereby enhancing mechanical responsiveness, agility, and reliability.

### 2.3 PFLA's Design Characterization

**Figure 3:**
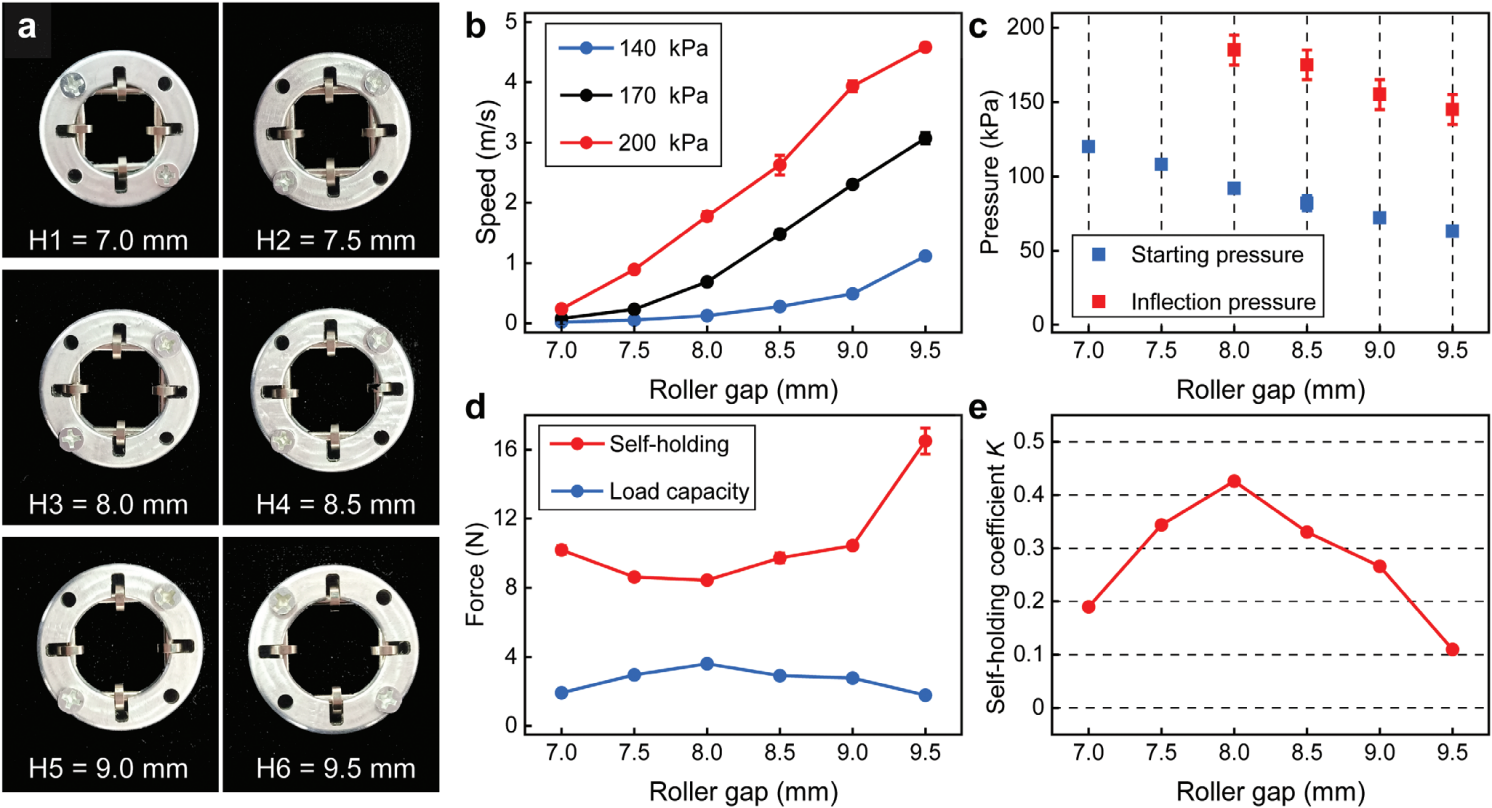
Actuation performance of the PFLA under different roller gaps. a) Pictures of sliders with varying roller gaps. b) Speeds of movable systems under input pressures of 140, 170, and 200 kPa. c) Starting pressure and inflection pressure of the movable system under various roller gaps. d) Quantified value for self‐holding and load capacity of sliders with various roller gaps. e) Self‐holding coefficient K for sliders with different roller gaps.

To investigate the effect of different parameters on the performance of the PFLA, sliders with varying roller gaps were fabricated and tested. The smaller the roller gap, the greater the deformation of the guide tube and the higher the rolling resistance of the slider. Conversely, the piston may pass through the slider with a larger roller gap, resulting in actuation failure. Therefore, to ensure effective actuation, an optimal roller gap range of 7–9.5 mm was determined, with the corresponding sliders labeled as H1 to H6 (**Figure** **3**a). These sliders were tested using input pressures ranging from 0 to 200 kPa (Table S1, Supporting Information). The driving speeds of the PFLAs at 140, 170, and 200 kPa are depicted in Figure 3b. The speed increased with the widening of the roller gap. The lowest maximum speed was observed at H1, measuring 0.24 m s^–1^, while the highest was at H6, reaching 4.58 m s^–1^. The smaller the roller gap, the higher the starting pressure and inflection pressure (Figure 3c). For the PFLAs utilizing sliders H1 and H2, their inflection pressures exceeded 200 kPa.

We also quantified the self‐holding and load capacity under various roller gaps. Theoretically, a smaller roller gap corresponds to a greater axial force component (Figure S1c, Supporting Information); as the roller gap decreases, the load capacity should increase, and the upper limit of the self‐holding force should decrease. However, there existed an intermediate value of roller gap (H3) that maximized the load capacity (3.6 N) and minimized the self‐holding force (8.4 N) (Figure 3d). It is attributed to the wrinkling of the guide tube caused by small roller gaps, which induces additional resistance to the movable system. The smaller the roller gap, the more pronounced the wrinkle effect. We defined a dimensionless coefficient, *K*, as the reliability coefficient of self‐holding, calculated as the load capacity ratio to the maximum self‐holding force. A smaller K value indicates better self‐holding reliability. With the increase in the roller gap, K first increased and then decreased, and reached the maximum at H3 (Figure 3e). Additionally, *K* was always below 0.5, indicating that the loaded PFLA, without input pressure, could maintain a steady state even when subjected to an external impact.

**Figure 4:**
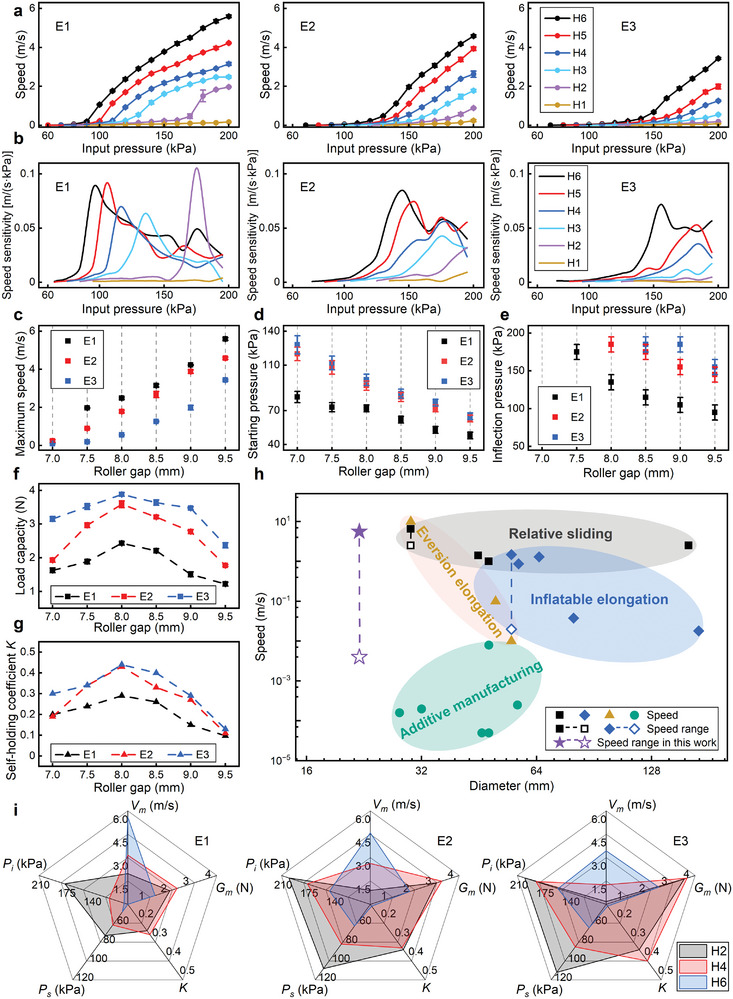
Design characterization of the PFLA. a) Speed and b) speed sensitivity for the PFLAs using tubes with E1, E2, and E3 under various sliders and input pressures. When using different roller gaps and elastic modulus, the PFLAs' c) maximum speed *V*_*m*_, d) starting pressure *P*_*s*_, e) inflection pressure *P*_*i*_, f) load capacity *G*_*m*_, g) self‐holding coefficient *K*. h) Comparison of the maximum radial dimension and motion speed of long‐distance linear robots utilizing different action mechanisms. i) Five key performance indexes (*P*_*s*_, *P*_*i*_, *V*_*m*_, *G*_*m*_, and *K*) for each PFLA using E1, E2, and E3, respectively, under sliders with roller gaps of H2, H4, and H6. In (a), (c), (d), (e), and (f), points are averages of three trials, and error bars show ± SD.

We subsequently evaluated the performance of the PFLA by employing three tubes with elastic modulus E1, E2, and E3 (E1 < E2 < E3). The tube previously used is E2. Figure S9 (Supporting Information) illustrates the response of three sealed tubes to input pressure. The speed (**Figure** **4**a) and speed sensitivity (Figure 4b) decreased with an increase in the elastic modulus of the guide tube. When employing the H6 slider, the maximum speeds for PFLAs using E1, E2, and E3 were 5.6, 4.58, and 3.4 m s^–1^, respectively (Figure 4c). The greater the elastic modulus of the tube, the larger the normal force acting on the piston, and the higher the input pressure needed to overcome moving resistance. Hence, both the starting pressure (Figure 4d) and the inflection pressure (Figure 4e) increased with the elastic modulus, and decreased with the roller gap. When employing the H1 slider, the starting pressure for PFLAs using E1, E2, and E3 were 82, 120, and 128 kPa, respectively. The inflection pressure of PFLA using H6 and E1 was the lowest, between 90 and 100 kPa. Data not in Figure 4e suggests the inflection pressure under the current parameters exceeds the tested pressure range. Additionally, guide tubes with a higher elastic modulus exhibit less deformation under pneumatic pressure, resulting in better load capacity (Figure 4f). The PFLA using an E3 tube demonstrated the highest load capacity, reaching a maximum of 3.9 N (H3 slider). The reliability coefficient *K* increased slightly with the increase in elastic modulus, and remaining consistently below 0.5 (Figure 4g). The measurement of the self‐holding capability is illustrated in Figure S10 (Supporting Information).

Five key performance indexes' starting pressure (*P*_*s*_), inflection pressure (*P*_*i*_), maximum speed (*V*_*m*_), load capacity (*G*_*m*_), and reliability coefficient (*K*)'are used to evaluate the performance of the PFLA using different guide tubes (E1, E2, and E3) and sliders (H2, H4, and H6), as illustrated in Figure 4i. The lower elastic modulus enhances the guide tube's sensitivity to pressure changes, broadening PFLA's speed modulation range and improving self‐holding reliability (*K*). Conversely, a higher elastic modulus offers increased resistance to pressure fluctuations, facilitating finer speed regulation and enhanced load capacity. For the roller gap, a larger value corresponds to a wider speed range, while a more minor one results in higher speed resolution. Figure 4h presents a diagram illustrating the maximum radial dimension and motion speed of long‐distance linear robots utilizing different action mechanisms, including relative sliding,^[^ ^33^, ^34^, ^36^, ^39^ ^]^ inflatable elongation,^[^ ^35^, ^40^, ^41^, ^42^, ^43^ ^]^ eversion elongation,^[^ ^26^, ^44^, ^45^ ^]^ and additive manufacturing.^[^ ^29^, ^30^, ^46^, ^47^, ^48^, ^49^ ^]^ The PFLA, based on the PPW principle in this study, features a compact structure and achieves a wide speed range from 0.004 to 5.6 m s^–1^ within the pressure range of 200 kPa. Furthermore, it incorporates two safety mechanisms'self‐protection and self‐holding'by leveraging the unique modulation of tube diameter and elasticity.

## 3 PFLA's Application

### 3.1 Wearable Device for Limb Assistance in Humans

**Figure 5:**
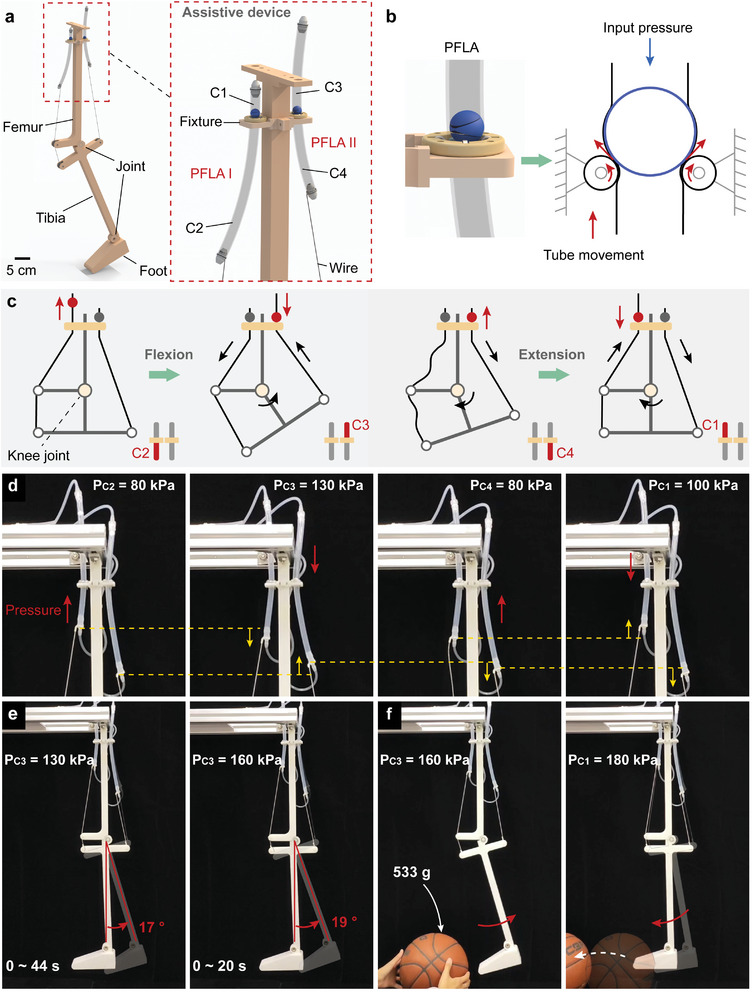
Application of wearable device for rehabilitating the human knee joint. a) A knee joint model equipped with the assistive device (inset: a magnified schematic of two PFLAs). The traction wire connects the end of the guide tube to the tibia, and the slider is affixed to the fixture, with the piston located above the slider. b) Installation and operating principle of the PFLA on the joint model. c) The fundamental motion of the knee joint within one flexion‐extension cycle and the corresponding driving signal. The red area denotes a pressurized chamber, while the grey denotes that the chamber is at atmospheric pressure. d) Variations in the positions of guide tubes I and II within one cycle (yellow arrow). e) Flexion of the knee joint under input pressures of 130 and 160 kPa. f) The joint model kicks a basketball with the assistance of the wearable device.

Wearable assistance devices are vital for aiding individuals with health issues in recovery and function enhancement. However, limitations such as bulky designs and lack of personalized adjustments restrict their versatility and may impact user experience negatively. We designed a lightweight assistive device (weighing a total of 27 g) for human limbs, consisting of two PFLAs, two inextensible traction wires, and a fixture (**Figure** **5**a; Figure S11, Supporting Information). Its functional effectiveness was demonstrated using a 3D‐printed joint model. PFLAs I and II were positioned at the front and rear of the joint model, respectively, to assist in extending and flexing the knee joint by simulating muscle traction. To balance moving resistance and load capacity, we determined the parameter settings of PFLAs I ([H5, E2]) and II ([H3, E2]). The movable system in this device was fixed, and it operated by utilizing the tension exerted by the guide tube to transmit force and displacement, rather than relying on the movable system. As depicted in Figure 5b, when pressure was applied to the upper chamber, the piston and the deformed tube wall exerted force and moment on the rollers of the slider, causing them to rotate. The rotating rollers, in turn, generated friction against the surface of the tube, urging it upward; the ascending guide tube provided tension for the tibia. The use of two such PFLAs allows for an antagonistic mechanism to assist knee joint flexion and extension.

The wearable device operates on the principle that before one PFLA applies a pulling force, the piston of the other PFLA must be released, ensuring that the self‐holding mechanism does not hinder joint rotation. Specifically, to initiate the flexion action, we started by pressurizing chamber C2 to raise piston I (Figure 5c). Subsequently, we pressurized chamber C3, causing the joint to flex as tube II was pulled upward. Thanks to the self‐holding mechanism, the knee joint maintained flexion even when the pressure in C3 was released. To initiate the extension action, we pressurized chamber C4 to elevate piston II, resulting in a partial extension of the knee joint under the influence of tibial gravity. Following this, we pressurized chamber C1 to enable complete joint extension through the traction applied via tube I. The device can assist in knee flexion‐extension training within 0–22°. Figure 5d shows the positional variations of guide tubes I and II throughout a single cycle. When the pressure in chamber C3 was set to 130 kPa, the joint flexed 17° in 44 s. To test the influence of pressure on flexion, we pressurized chamber C3 by 160 kPa. As a result, the flexion speed of the knee joint accelerated, reaching 19° within 20 s (Figure 5e). The speed of knee flexion transitioned from fast to slow because as the joint rotated, the direction of tension changed, leading to an increase in the force required for knee flexion as the degree of flexion increased. When the required force increased to equal the load capacity of the PFLA, the joint reached its maximum flexion angle. We also demonstrated that the joint model kicked a 533 g basketball with the assistance of this device (Figure 5f; Movie S6, Supporting Information). The proposed PFLA is lightweight, flexible, and well‐suited for the human body, with the potential to serve as a high‐performance assistance device. Moreover, the equipment can automatically lock during a power outage or malfunction, ensuring safety and controllability.

### 3.2 Rotary Joint Module for Manipulators

**Figure 6:**
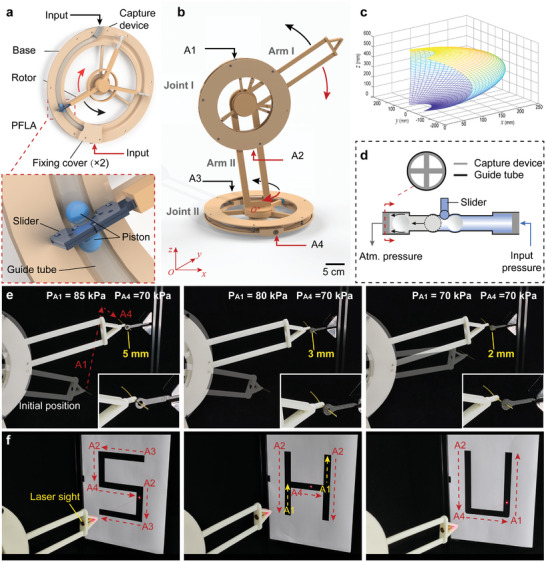
Precise operation of the manipulator with two rotary joints. a) The composition and operational principles of the rotary joint module. b) The manipulator using two rotary joints. Joint I has two pressure supply ports, A1 and A2, and Joint II has two air pressure supply ports, A3 and A4. c) Workspace of the manipulator's end. d) Rigid piston capture device. Both ends of the guide tube have capture devices and fixing covers. e) Manipulator guides the wire through holes with diameters of 5, 3, and 2 mm, respectively. The dashed lines indicate the trajectories of the manipulator under input pressure, and the port numbers in the figure represent pressurized ports. f) Using the end of the manipulator, draw the letters ‘S’,‘H’, and ‘U’ on the board, respectively.

Rotary manipulators are widely used for precise positioning and operation in three‐dimensional space. Utilizing the flexibility and high transmission precision of the proposed PFLA, we designed a rotary joint module capable of accurate positioning. The rotary joint was mainly composed of a base, a rotor, and a PFLA (**Figure** **6**a). The guide tube (E2 tube) of the PFLA was fitted within the groove of the base, and both ends were fixed. The slider was redesigned and integrated with the rotor, enabling it to push the rotor for rotation around the base center during actuation. To demonstrate the practical application of the joint module, we designed and manufactured a manipulator with two rotary joints (Figure 6b; Figure S12, Supporting Information). The two joints operated in mutually perpendicular planes, and each joint had a rotational range of 140°. Figure 6c depicts the workspace of the developed manipulator's end. To achieve bidirectional actuation, two pistons were employed and positioned on opposite sides of the slider. In addition, we designed a rigid piston capture device with an inner diameter of 11 mm and positioned it between the guide tube and the input tube (Figure 6d). When pressurizing, the sealed chamber expands, allowing the input air to pass over the first piston and form a pressure chamber between the two pistons. The second piston is first pushed into the capture device, and simultaneously, this pressure chamber is connected to atmospheric pressure. By applying air pressure to different ports, the joint can rotate both clockwise and counterclockwise directions.

To show the precise operational capabilities of the manipulator, we used it to guide a wire through a target hole. Fixing a wire to the end of the manipulator, we initially positioned it diagonally below the target hole. First, pressure (*P*_*A*1_) was applied to port A1 to drive joint I, rotating arm I counterclockwise. Then, *P*_*A*4_ was applied to port A4 to initiate clockwise rotation in joint II, enabling the wire to pass through the target hole. The manipulator successfully guided the wire through holes with diameters of 5, 3, and 2 mm, respectively (Figure 6e). Moreover, to demonstrate the trajectory‐tracking performance of the manipulator, a laser sight was attached to its end for path visualization. By regulating the air pressure signals, the manipulator successfully traced the letters “S,”“H,” and “U” on the board (Figure 6f; Movie S7, Supporting Information). This pneumatic manipulator achieves lightweight and energy‐efficient operation by externalizing power components. Additionally, lacking motors or electronic components, the device can operate effectively in high temperatures, explosive environments, or conditions potentially harmful to electrical systems.

### 3.3 Versatile Applications in Challenging Environments

**Figure 7:**
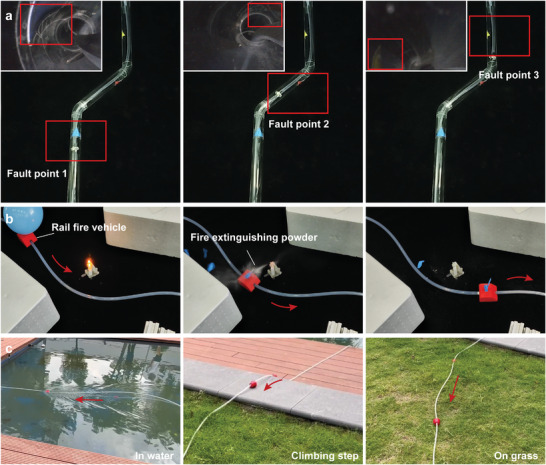
Versatile applications in challenging environments. a) The PFLA carries a miniature camera for pipeline inspection. b) The PFLA carries fire extinguishing powder to patrol and extinguish fires. c) Operation in various environmental conditions, including water, steps, and grassy areas.

The PFLA excels as a guideway robot in complex environments. Its high‐speed capability ensures rapid movements, enhancing efficiency in repetitive tasks. Furthermore, the proposed PFLA can achieve sensitive speed modulation by varying air pressure, especially at higher speeds. This ability is vital for applications requiring real‐time adjustments, such as search and rescue operations. Additionally, with its impressive adaptability and compact design, the PFLA demonstrates significant potential as an effective tool for navigation and operation in challenging environments. We demonstrated the potential of the PFLA in various scenarios, including its capability for inspections in confined environments. Using a pipeline with a 30 mm inner diameter and two 135° angles (**Figure** **7**a), we showcased its effectiveness. The PFLA's guide tube navigated curved pipelines through passive deformation under external force. Following this, a slider with a miniature camera was deployed for pipeline wall inspection. The device successfully detected the three preset fault points. For more complex environments requiring regular inspections, the guide tube can be pre‐positioned using methods such as drones and mobile robots, allowing for straightforward periodic checks thereafter. We conducted a cyclic test on the actuator, with detailed results provided in the Supporting Information (Figure S14). Additionally, the PFLA can patrol along a preset path. We simulated a fire scene using burning foam. As illustrated in Figure 7b and Movie S8 (Supporting Information), the slider carried fire extinguishing equipment (balloons with fire extinguishing powder) and successfully extinguished the fire. Because the PFLA's driving medium is isolated from the working environment, it can operate in various environmental conditions, including water, steps, and grassy areas (Figure 7c; see Figure S13, Supporting Information, for design details). In this outdoor experiment, we used a portable hand‐operated air pump to drive the PFLA without electrical equipment. Additionally, the proposed PFLA can easily increase the actuation distance by simply extending the guide tube. We tested the response time at driving distances of 1 and 20 m under varying air pressures.The results indicate that extending the distance by 19 m led to a maximum increase in response time of 0.59 s, as detailed in Figure S15 (Supporting Information). Therefore, for general or shorter travel distances, the response time is minimally affected. For longer tubing lengths, the trade‐off between actuation distance and response time appears to be acceptable.

## 4 Discussion

In this study, we introduce the PFLA, a flexible linear actuator for long‐distance and high‐precision applications. Inspired by the body size adjustment observed in snake swallowing, the PFLA utilizes pneumatic pressure to simultaneously drive a spherical piston and adjust the diameter of the tube to modify friction resistance. Relying on this combined mechanism of drive force and moving resistance adjustment, the PFLA possesses three excellent features, including self‐holding at rest, a wide speed regulation range from micro (0.004 m s^–1^) to macro (5.6 m s^–1^), and a self‐protection function based‐on pressure release. The primary objective of PFLA development is to create a flexible transmission device that can modulate speed over a wide range at relatively low pneumatic pressures while incorporating safety mechanisms. Flexible systems achieve smoother and more accurate trajectories through passive deformation compared to rigid systems. Compared to traditional rigid actuators like screw slides, the PFLA exhibits the capability to transmit along curved paths, a challenge for conventional rigid devices. In contrast to muscle‐type pneumatic artificial muscles, its actuation distance can be easily adjusted by changing the tubing length, rendering it more suitable for designing compact and integrated robotic systems. The PFLA's effectiveness in flexible and rigid systems was verified via a wearable device assisting the knee joint and a rotary joint module, respectively.

Existing long‐distance actuators have shown outstanding speed characteristics (Figure 4h).^[^ ^26^, ^34^ ^]^ The growth‐type robot mentioned in ref. [26] reaches a top speed of 10 m s^–1^ and can navigate various environments. However, this type of actuator is a standalone device, not designed for integration into different mechanical devices for propulsion. In contrast, our PFLA can be integrated into a wide variety of devices, offering broader applicability. The actuator described in ref. [34] displays favorable speed characteristics, with speeds ranging from 2 to 6.5 ms^–1^, but achieving precise positioning is challenging. Moreover, it requires a higher working pressure, ranging from 250 to 500 kPa, a level difficult for small portable air pumps to reach. In contrast, the developed PFLA, although its maximum speed of 5.6 m s^–1^ is lower than that mentioned in ref. [34], offers superior low‐speed performance, crucial for precise positional control. Additionally, reaching its maximum speed requires less than 200 kPa of air pressure, which is manageable with portable pumps. Moreover, the PFLA is equipped with self‐holding and self‐protection mechanisms, making it suitable for a wider range of applications. Utilizing widely produced tubes, the PFLA has a low production cost, requiring only $0.16 to extend the actuation distance by one meter. The PFLA, unlike electrically driven devices, does not need intricate waterproofing and dustproofing, making it suitable for various outdoor environments like water and grasslands. Additionally, the PFLA's low air consumption and driving pressure are facilitated by its lack of circulating inflation and deflation needs and small tube diameter. A manual portable air pump suffices for driving the PFLA, especially useful in limited power supply scenarios. Due to its excellent characteristics, low manufacturing costs, simple structure, and user‐friendly features, the PFLA holds great potential for application in various types of robots or mechanical devices. Future research could consider incorporating flexible sensing technology to endow the PFLA with additional capabilities and achieve more precise speed control through a closed‐loop feedback system. Additionally, exploring the parallel use of multiple PFLAs to realize richer functionalities is also a valuable research direction.

## 5 Experimental Section

### Materials and Fabrication of PFLA

The PFLA consisted of a spherical piston, a soft guide tube, and a wheeled slider. A polytetrafluoroethylene (PTFE) ball (Φ9 mm, Shanghai Xin Wang Rubber & Plastic Hardware Store) was used as the piston. A ready‐made silicone tube (8 mm inner diameter and 10 mm outer diameter, Shanghai Shenxin rubber products factory) was used as the guide component. As shown in Figure S4 (Supporting Information), the PFLA's slider comprised two CNC‐machined aluminum alloy sheets (Wuhan Pilot 3D Technology Co., Ltd) and four rollers. The four rollers were positioned within grooves on these sheets. Each roller was composed of a miniature bearing (1.5‐mm‐inner diameter, 4‐mm‐outer diameter, 1.2‐mm‐thickness, Shanghai Yanxu Industry Co., Ltd) and a miniature steel shaft (Φ1.5 mm, Shenzhen Mingchen Metal Material Store). The two aluminum alloy sheets were connected by screws (M2, Shenzhen Bairuite Fastener Co., Ltd).

### Data Acquisition and Analysis

ORIGIN was used to visualize the experimental data, and the speed sensitivity data were smoothed using the “Modified Bezier curve.” In some experiments, the measured values either increased asymptotically to a stable value or exhibited a small oscillatory component. In all cases, the recorded value was the average data from the stable region of the system.

### Finite Element Simulation of the PFLA

The fluid‐solid coupling module of COMSOL Multiphysics was employed for simulation. In the simulation model, the diameter of the guide tube's inlet was set to be larger than that of the piston, ensuring a smoother entry of the piston into the channel. To enhance the tractability and computational efficiency of analysis, the PFLA's slider was modeled as a ring with an inner diameter matching the roller gap, and sliding friction with a low coefficient was used to replace rolling friction.

## Conflict of Interest

The authors declare no conflict of interest.

## Supporting information

Supporting Information

Supporting Movie S1

Supporting Movie S2

Supporting Movie S3

Supporting Movie S4

Supporting Movie S5

Supporting Movie S6

Supporting Movie S7

Supporting Movie S8

## Data Availability

The data that support the findings of this study are available from the corresponding author upon reasonable request.
