# Supplementary material for: A Pneumatic Flexible Linear Actuator Inspired by Snake Swallowing
Source: Adv Sci (Weinh). 2024 Sep 12;11(41):2405051. doi: 10.1002/advs.202405051 (PMC11538662; doi:10.1002/advs.202405051)
Supplement: Supplementary file 1 — Supporting Information [file ADVS-11-2405051-s002.docx]

**Supplementary Information**

**A pneumatic flexible linear actuator inspired by snake swallowing**

Yuyan Qi†, Jiaqi Shao†, Yongjian Zhao, Tong Niu, Yi Yang, Songyi Zhong, Shaorong Xie, Yangqiao Lin*, Yang Yang*

*Corresponding author, Email: [linyangqiao@shu.edu.cn (Y. L.);](mailto:lyq627@zju.edu.cn,) [yangyang_shu@shu.edu.cn](mailto:yangyang_shu@shu.edu.cn) (Y. Y.)

† These authors contributed equally to this work.

Supplementary Information contains:

Notes S1-S5

Figures S1-S18

Table S1

Movies S1-S8

**Supplementary Text**

**Note S1. Mentioned definitions.**

Self-holding: When there is no pressure input, the deformed guide tube exerts a significant normal force on the piston surface, firmly anchoring it within the airflow channel. This mechanism is referred to as self-holding.

Self-holding capacity: The minimum force required to induce reverse movement of the movable system without applying pressure, numerically equal to the upper limit of the self-holding force.

Self-protection: At higher pressures or in overload conditions, the inner diameter of the sealed chamber in the guide tube expands beyond the diameter of the piston, forming a gap between the inner wall of the guide tube and the piston. This allows some air to be released into the atmosphere, reducing the chamber's internal pressure and limiting the driving force acting on the piston. This mechanism is referred to as the PFLA's self-protection. In an overload scenario, regardless of how high the air pressure is, the movable system cannot start.

Load capacity (*G_m_*): The maximum load that the movable system can effectively drive.

Self-holding coefficient (*K*): A dimensionless coefficient used to evaluate the reliability of self-holding, calculated as the ratio of load capacity to the upper limit of the self-holding force. The smaller the *K* value, the better the reliability of self-holding.

Starting pressure (*P_s_*): The minimum input pressure required to move the movable system.

Maximum speed (*V_m_*): The speed at an input pressure of 200 kPa.

Inflection pressure (*P_i_*): The input pressure corresponding to the maximum speed sensitivity.

**Note S2. Modeling the force of the movable system**

We model the PFLA utilizing force balance in a quasi-static state, describing how generated force varies with input pressure^1,2^. The model is based on four key assumptions: (i) The friction between the rollers and the tube surface is large enough, so the rollers only roll along the guide tube without sliding. (ii) The deformation of the guide tube always follows Hooke’s law. (iii) When pressurized, no deformation occurs in the axial direction. (iv) The mass of the PFLA can be negligible. Here, we model a 2D axial section of the PFLA and assume that this section undergoes continuous rotation of 360 degrees along its central axis. (Figure S1a). We parameterize the PFLA’s shape by defining various variables: *R_pre_* represents the inner radius of the sealed chamber. *R_pis_* and *R_rol_* are the radius of the piston and the rollers, respectively. *R_tube_* is the inner radius of the guide tube, *H* is the roller gap, and *T_tube_* is the initial wall thickness of the guide tube. When there is no pressure input, *R_pre_* is equal to *R_tube_*. As shown in Figure S1b, the axial force balance of the roller is as follows.

$F_{thr}\sin\phi_{1}+F_{f\_rol}=F_{gen}+F_{ten}\cos\phi_{1}$ (S1)

Based on the moment balance, the following equation can be derived:

$F_{ten}R_{rol}=F_{f\_rol}R_{rol}$ (S2)

Where *F_ten_* is the tension of the deformed tube wall. *F_thr_* represents the normal force acting on the roller, directed from the center of the piston towards the center of the roller. *F_f_rol_* is the tangential force acting on the roller surface. We neglect the deformation of the guide tube in the contact area with the roller and take point A as its action point. *F_gen_* is derived from the balance of forces applied to the rollers. *φ_1_* is the angle between the line connecting the piston center and the roller center and the vertical direction.

$\phi_{1}=\arccos(\frac{R_{rol}+\frac{H}{2}}{R_{pis}+R_{rol}+T_{tube}})$ (S3)

For *R_pre_* < *R_pis_*, the tension is primarily generated by the piston. We use the differential element method to solve *F_ten_*, as shown in Figure S1d. According to Hooke’s law, the tension of an infinitesimal element is expressed as:

$dF_{ten}=E\frac{L'-L}{L}dS_{1}$ (S4)

Where *E* is the elastic modulus of the guide tube, *L* and *L*' represent the lengths of the tube section in contact with the piston before and after deformation, respectively. *dS_1_* is the cross-sectional area of the infinitesimal element.

$\begin{matrix} L=2R_{pis}\sin(\frac{\phi_{1}+\phi_{2}}{2}) \\ L'=R_{pis}\text{（}\phi_{1}+\phi_{2}\text{）} \end{matrix}$ (S5)

$dS_{1}=\frac{1}{2}[(R_{pis}+T_{tube})^{2}-R_{pis}^{2}]d\theta$ (S6)

Where *φ_2_* is the contact angle between the piston and the guide tube on the driving side:

$\phi_{2}=\arccos\frac{R_{pre}}{R_{pis}}$ (S7)

*dθ* is the central angle corresponding to the infinitesimal element, we can get:

$F_{ten}=\frac{1}{2}\int_{0}^{2\pi} E\frac{L^{'}-L}{L}[(R_{pis}+T_{tube}')^{2}-R_{pis}^{2}]d\theta$ (S8)

For *R_pre_* ≥ *R_pis_*, the tension is primarily generated by the input pressure. The equation can be obtained based on the balance between axial stress in the guide tube wall and pressure on the internal cross-section:

$\pi[(R_{pre}+T_{tube}')^{2}-R_{pre}^{2}]\sigma_{a}=P\pi R_{pre}^{2}$ (S9)

Where *σ_a_* is the axial stress of the deformed tube, *P* is the input pressure. Axial strain and the smaller radial strain are negligible. The uniformly expanded tube can be considered as a thin-walled elastic hollow cylinder under pressure. According to Laplace’s equation:

$P=\frac{\sigma_{\theta}T_{tube}'}{R_{pre}}$ (S10)

Where *σ_θ_* represents the circumferential stress of the tube wall, which is determined by applying Hooke’s law in three-dimensional space.

$\sigma_{\theta}=\frac{R_{pre}-R_{tube}}{R_{tube}}(\frac{E}{1-\nu^{2}})$ (S11)

Where *ν* is the Poisson’s ratio of the tube. As the volume of the tube remains unchanged before and after deformation, we can deduce:

$T_{tube}'=\frac{R_{tube}}{R_{pre}}T_{tube}$ (S12)

Substitute *σ_θ_* and *T_tube_*' in equation (S10) with the equations (S11) and (S12), respectively.

$P=\frac{R_{pre}-R_{tube}}{R_{pre}^{2}}(\frac{E}{1-\nu^{2}})T_{tube}$ (S13)

As shown in Figure S1c, the axial force acting on the piston can be obtained as follows.

$F_{pre}=F_{thr}'\sin\phi_{1}+F_{f\_pis}$ (S14)

*F_thr_*' represents the reaction force corresponding to *F_thr_*. *F_pre_* is generated by input pressure, and *F_f_pis_* is the maximum static friction between the piston and the tube wall. *F_pre_* can be expressed as:

$\left\{\begin{matrix} F_{pre}=\pi PR_{pre}^{2}(R_{pre}<R_{pis}) \\ F_{pre}=\pi PR_{pis}^{2}(R_{pre}\geq R_{pis}) \end{matrix}\right.$ (S15)

Where *F_f_pis_* is equal to the friction coefficient *μ* times the normal force *F_N_* generated by the deformed tube wall on the piston.

$\left\{\begin{matrix} F_{f\_pis}=\mu F_{N} \\ F_{N}=F_{N1}+F_{N2} \end{matrix}\right.$ (S16)

The tube wall is deformed circumferentially and axially at the part in contact with the piston. As illustrated in Figure S1d, the circumferential stress of the deformed tube wall is determined through calculus, where each differential element exerts a force of *P_θ_*.

$P_{\theta}=\frac{R_{pis}\sin\phi-R_{tube}}{(R_{pis}\sin\phi)^{2}}(\frac{ET_{tube}}{1-\nu^{2}})$ (S17)

The cross-sectional area *dS_2_* is expressed as:

$dS_{2}=2\pi R_{pis}\sin\phi R_{pis}d\phi\sin\phi=2\pi R_{pis}^{2}\sin^{2}\phi d\phi$ (S18)

The *F_N1_* is given by:

$F_{N1}=\int_{\frac{\pi}{2}-\phi_{1}}^{\frac{\pi}{2}+\phi_{2}} P_{\theta}2\pi R_{pis}^{2}\sin^{2}\phi d\phi$ (S19)

The normal force *F_N2_* generated by axial deformation can be calculated as follows.

$F_{N2}=F_{ten}'(\sin\phi_{1}+\sin\phi_{2})$ (S20)

*F_N_* is equal to *F_N1_* + *F_N2_*, by substituting equations S19 and S20 for *F_N1_* and *F_N2_* in S16, respectively, we can obtain:

$F_{f\_pis}=\mu\left[2\pi(\frac{ET_{tube}}{1-\nu^{2}})\int_{\frac{\pi}{2}-\phi_{1}}^{\frac{\pi}{2}+\phi_{2}} (R_{pis}\sin\phi-R_{tube})d\phi+F_{ten}'(\sin\phi_{1}+\sin\phi_{2})\right]$ (S21)

*F_f_H_* is the rolling resistance encountered by the roller during movement, which increases as the roller gap decreases. It can be expressed as follows.

$F_{f\_H}=\gamma\text{（}R_{tube}-\frac{H}{2}\text{）}$ (S22)

Considering the movable system as a whole, we get:

$F_{gen}=F_{pre}-F_{f\_pis}-F_{f\_H}$ (S23)

**Note S3. Modeling self-holding of the movable system.**

In addition to calculating the generated force, the same shape parameterization also allows an estimation of the self-holding force of the movable system. Assuming a force, *F_active_*, acts on the slider, and the movable system of the PFLA is in a static critical state (Figure S2). The force balance equation of the movable system is given as follows.

$F_{active}=F_{f\_pis}+F_{f\_H}+\mu F_{N}'$ (S24)

Here, we considered the influence of *H* on the resistance, *F_f_H_*, in actual operation. The contact angle between the tube and the piston is 2×*φ_1_*, symmetrically above and below the piston’s center. So we get the friction:

$F_{f\_pis}=\mu\left[2\pi(\frac{ET_{tube}}{1-\nu^{2}})\int_{\frac{\pi}{2}-\phi_{1}}^{\frac{\pi}{2}+\phi_{1}} (R_{pis}\sin\phi-R_{tube})d\phi+2F_{ten}'(\sin\phi_{1})\right]$ (S25)

Additionally, due to the function of *F_c_*, there is an additional normal force, *F_N_* ':

$F_{N}'=F_{thr}\cos\phi_{1}$ (S26)

Where *F_c_* can be determined as follows:

$F_{thr}=\frac{F_{active}}{\sin\phi_{1}}$ (S27)

**Note S4. Computing the model.**

We utilized MATLAB to calculate the generated force and self-holding force of the PFLA. Figures S3a and S3b illustrate how the generated force of the PFLA varies with input pressure. The results show some deviation between the model-predicted values and the actual measurements. As the air pressure increases, the predicted generated force increases approximately, while the actual measured values show a slightly slowing trend. Figure S3c shows the self-holding force of the PFLA with different roller gaps and elastic moduli. The predicted self-holding force increases with the roller gap, whereas the measured values indicate a roller gap that minimizes self-holding force. This is due to the additional resistance from the guide tube's wrinkling effect on roller movement. The model approximately captures the shapes of the generated force and self-holding force curves, despite slight deviations. These deviations might arise from two main factors. 1)The PFLA exhibited more complex behavior during actual operation, including intricate wrinkle deformations of the guide tube when using small roller gaps, and fluid resistance caused by air release. Simplifications were made in the mathematical model for mechanical analysis, which may not fully capture the complex deformations in the real system. 2) The material parameters in the model may differ from the actual material characteristics. While more precise models could potentially yield more accurate predictions, the current model captures the trends and approximate magnitudes of the forces, which are sufficient for design purposes.

**Note S5. Finite element method simulation details.**

We used COMSOL to simulate the transient movement of the piston within the guide tube under pneumatic pressure. A 2D axisymmetric model was established. The slider was simplified to a ring with an outer diameter of 16.5 mm and an inner diameter of 8.5 mm. The inner and outer diameters of the guide tube were set at 8 mm and 10 mm, respectively, while the piston diameter was set at 9 mm. Since the piston diameter exceeded the guide tube's inner diameter, the guide tube entrance was designed with a conical opening to facilitate the piston's entry under pneumatic pressure. The friction coefficients for the slider and piston were set to 0.02 and 0.1, respectively. The guide tube was modeled as a linear elastic material with a Young's modulus of 3.0 MPa and a Poisson's ratio of 0.48. Both the piston and slider were treated as rigid materials. The air density and viscosity were set to 1.02 kg/m³ and 1.75e-5 Pa·s, respectively. The maximum and minimum mesh sizes were set to 0.46 mm and 0.013 mm, respectively.

During the simulations, we used COMSOL’s built-in Laminar Flow Interface and Solid Mechanics Interface, and the Arbitrary Lagrangian-Eulerian (ALE) method to describe the deformation of the guide tube. As shown in Figure S5a, the simulations were completed in two steps:

Step I: Simulating the deformation caused by the slider fitting onto the guide tube. The flow field module was disabled in this step. At the initial moment of this step, the slider's diameter exceeded the guide tube's outer diameter, with no contact between them, resulting in no deformation of the guide tube. The slider diameter was then gradually reduced to the predetermined position, allowing it to fit onto the guide tube, and the guide tube deformation was calculated.

Step II: Using the geometry obtained from Step I as the initial value, the movement of the piston under pneumatic pressure was calculated. As the fluid medium was air, the flow was considered compressible, with inlet and outlet pressures set to 90 kPa and 0 kPa, respectively. All wall conditions were no slip. The parallel sparse direct solver MUMPS was used to solve the system of linear equations, and the backward differentiation formula (BDF) was chosen as the time-stepping method. Each time step was automatically determined by the software based on the residual in the last iteration.

**Supplementary Figures**


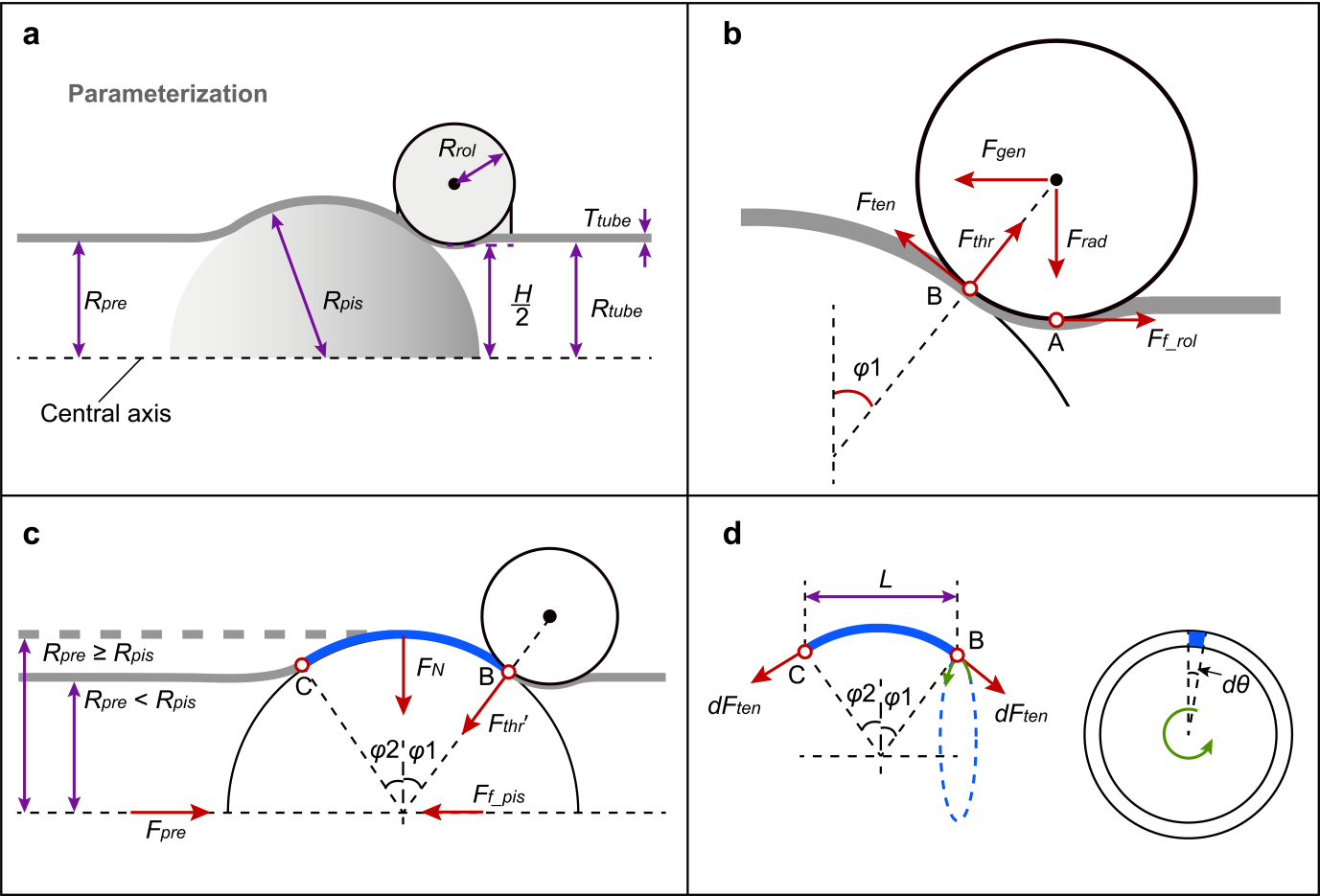


**Figure S1 | Force analysis for the PFLA under the input pressure. a,** Parameterization of the PFLA’s shape. **b** and **c,** Force analysis for the roller (b) and the piston (c). **d,** Analysis for circumferential force of the deformed guide tube.


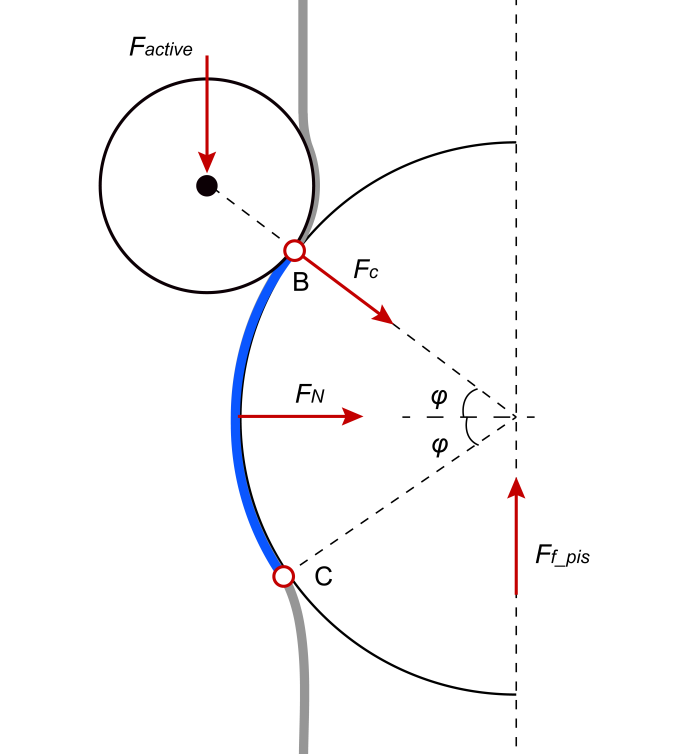


**Figure S2 | Force analysis for the PFLA without input pressure.** Points B and C represent the boundary of the tube in contact with the piston.

**Figure S3 | Comparison between model-predicted and actual measured values. a,** The generated force of the movable system was tested at various pressures and roller gaps, with the elastic modulus of the guide tube set to 2.5×10^6 Pa. **b,** The generated force of the PFLAs was tested with different tube elastic moduli (E1, E2, and E3) under varying air pressures. The roller gap of the slider was 8.0 mm (H3). **c,** Results for the self-holding force of the PFLAs using E1, E2, and E3 tubes under various roller gaps.


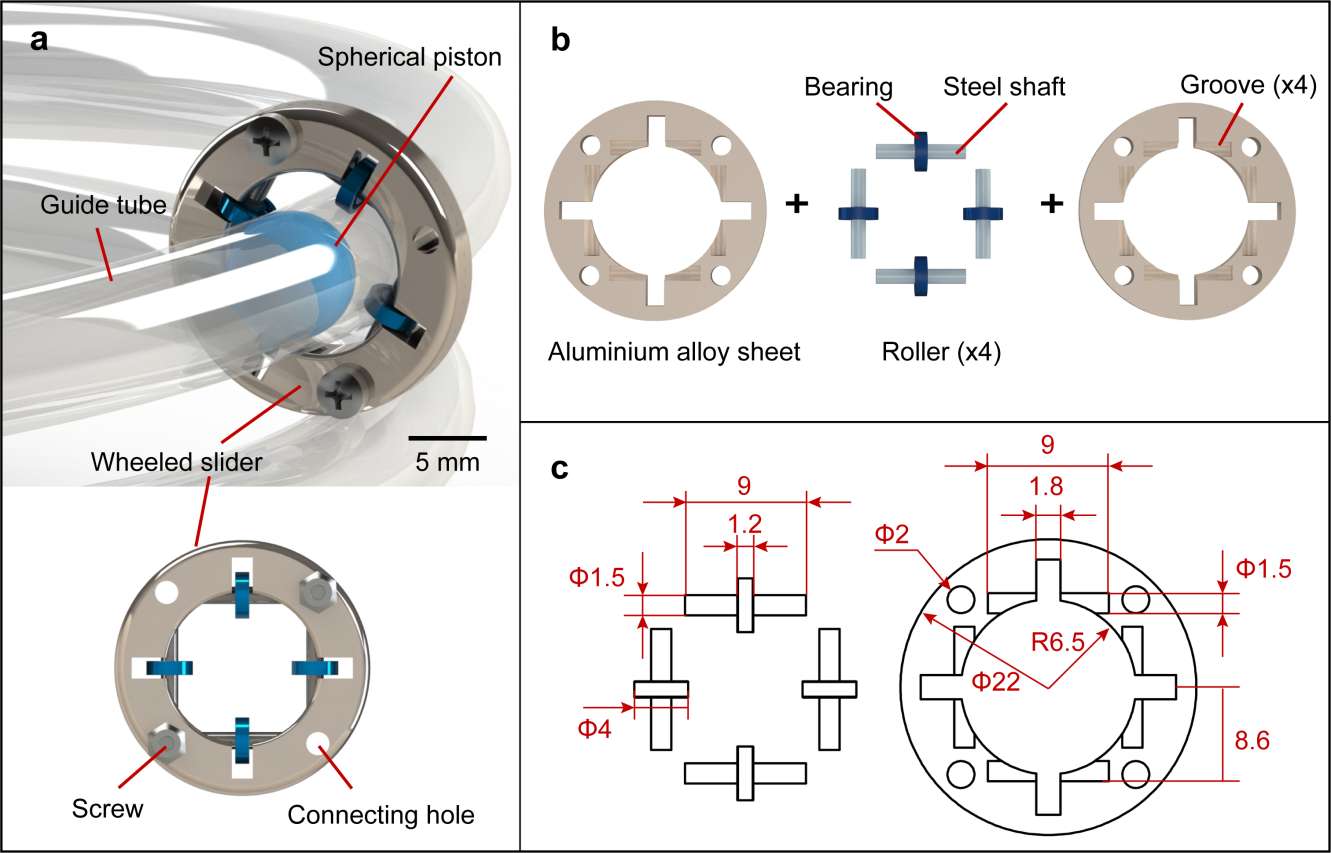


**Figure S4 | Schematic drawing of the typical PFLA. a,** Components of the PFLA. **b** and **c,** Assembly (b) and dimensions (c) of the slider.

**
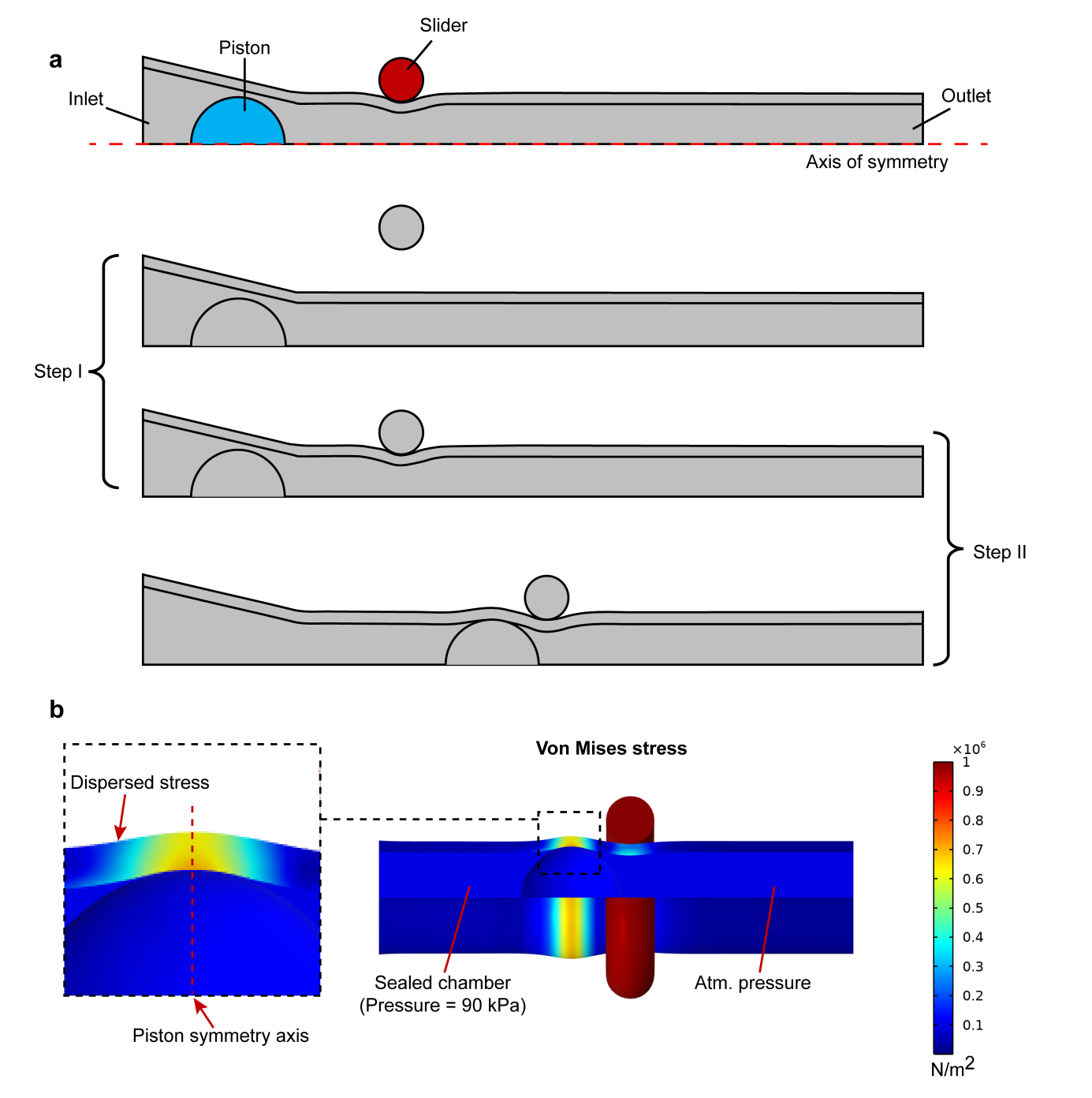
**

**Figure S5 | Finite element method simulation. a,** 2D axisymmetric model. **b,** Von Mises stress distribution in the guide tube. A half-section view of the PFLA is displayed for clarity. The figure illustrates the stress distribution of the tube wall within the finite element model when the pressure in the sealed chamber is 90 kPa. The expansion of the sealed chamber disperses the stress in the tube wall near the piston, resulting in a reduction of the normal force acting on the piston. This effect becomes more pronounced as the expansion of the sealed chamber intensifies.


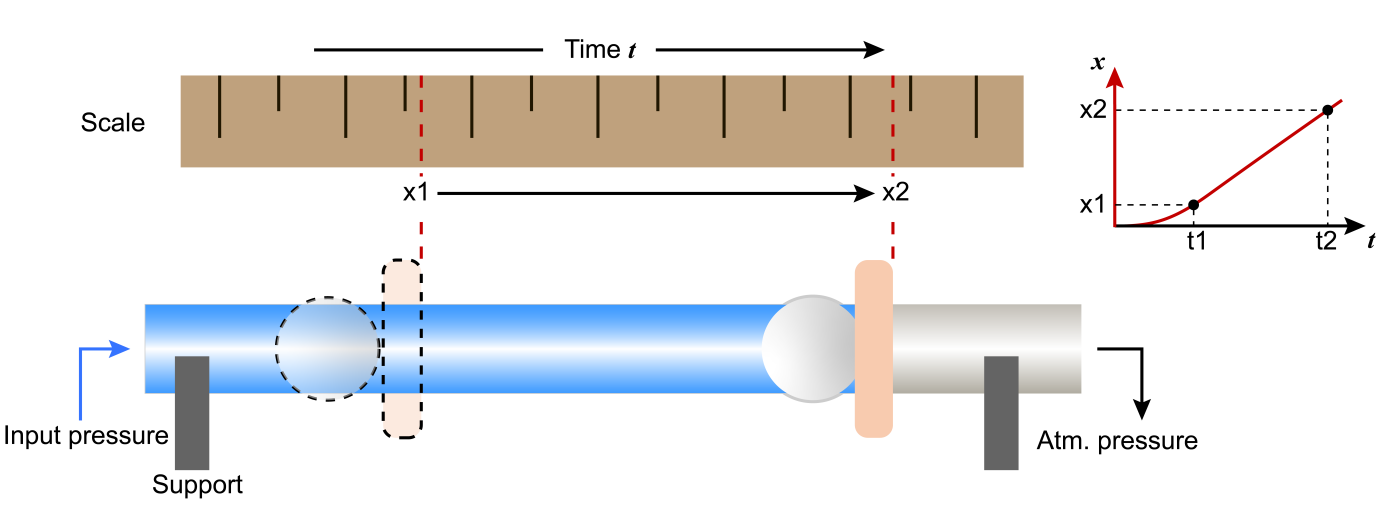


**Figure S6 | Speed measurement.** Due to the medium in the guide tube being uniform, the movable system can move at a constant speed along the tube under a constant input pressure. Two supports were used to elevate and stabilize the guide tube, preventing the slider from colliding with the environment. A measuring scale was positioned parallel to the guide tube above the PFLA to serve as a position reference for the movable system. Displacement of the movable system over time was recorded using a high-speed camera. When connected to a constant pressure source, the movable system initially accelerated briefly and then maintained a constant speed. The ratio of displacement to time at a constant speed (∆*x*/∆*t*, ∆*x* always takes 120 cm) is the average speed under this pressure.


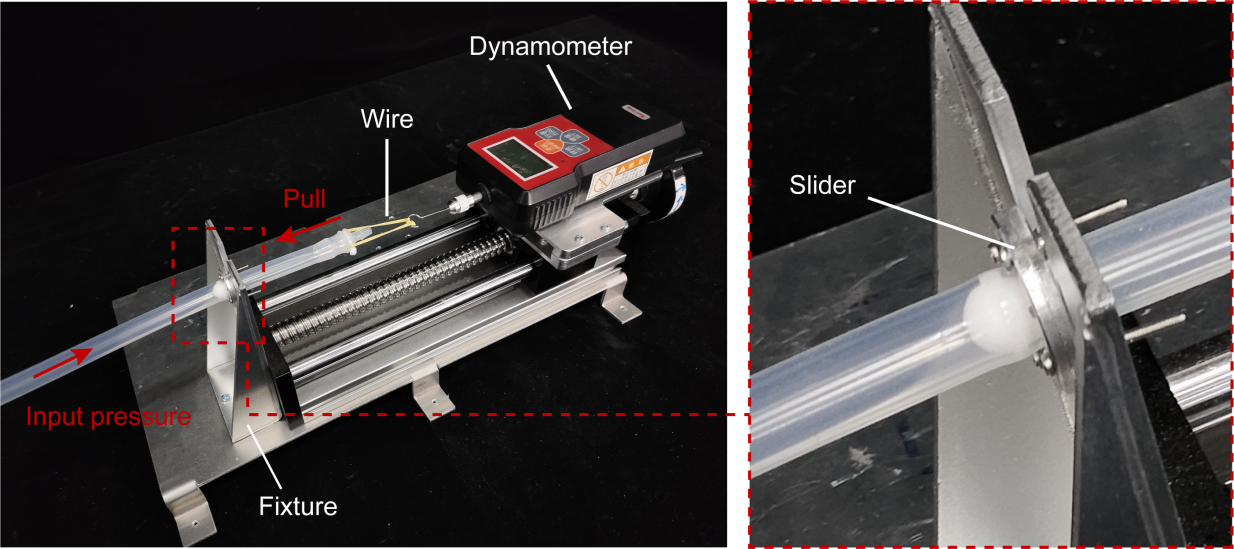


**Figure S7 | Experimental setup for load capacity measurement.** The slider was fixed on the fixture. An inextensible wire connected the end of the guide tube to the dynamometer. The centers of the slider, guide tube, and dynamometer were aligned in a straight line. Due to the slider being affixed, the guide tube will exert a pulling force on the dynamometer when pressurized. During the measurement, the input pressure was gradually increased from 0 to 200 kPa, and the maximum value displayed by the dynamometer was recorded as the load capacity of the PFLA.


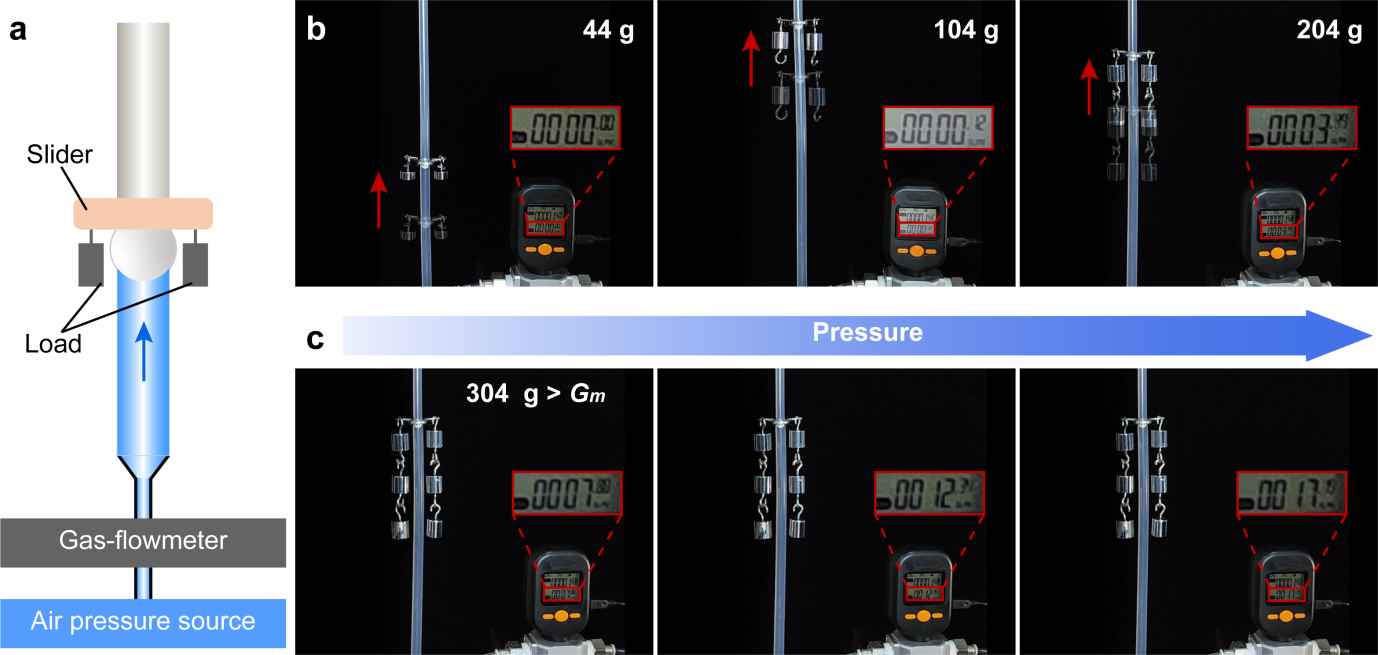


**Figure S8 | Measurement of air release from sealed chamber under various loads. a,** Experimental setup. The guide tube was vertically suspended, and a gas flowmeter was connected to the input port of the PFLA. **b,** The air release in the sealed chamber was measured under a constant pressure of 140 kPa while applying loads of 44, 104, and 204 g (all less than the upper load limit of 2.9 N). The results demonstrated that the released air increased with the load. Additionally, the movable system moved normally. **c,** The air release was tested under a load exceeding the upper limit of 2.9 N, with a value of 304 g. By increasing the input pressure from 140 to 200 kPa, we observed a gradual increase in the amount of released air, with no change in the slider’s position.

**Figure S9 | Outer diameter measurement of three sealed tube under various input pressures. a,** The guide tube was placed horizontally on a flat surface. The input pressure was increased from 0 to 200 kPa at intervals of 10 kPa, and the outer diameter of the sealed chamber was measured using a laser displacement sensor. **b,** Outer diameters of the three tubes (E1, E2, and E3) under different input pressures. Symbols represent the average values of three measurements, and the fitted line is obtained by linear fitting with a fixed intercept of 10.


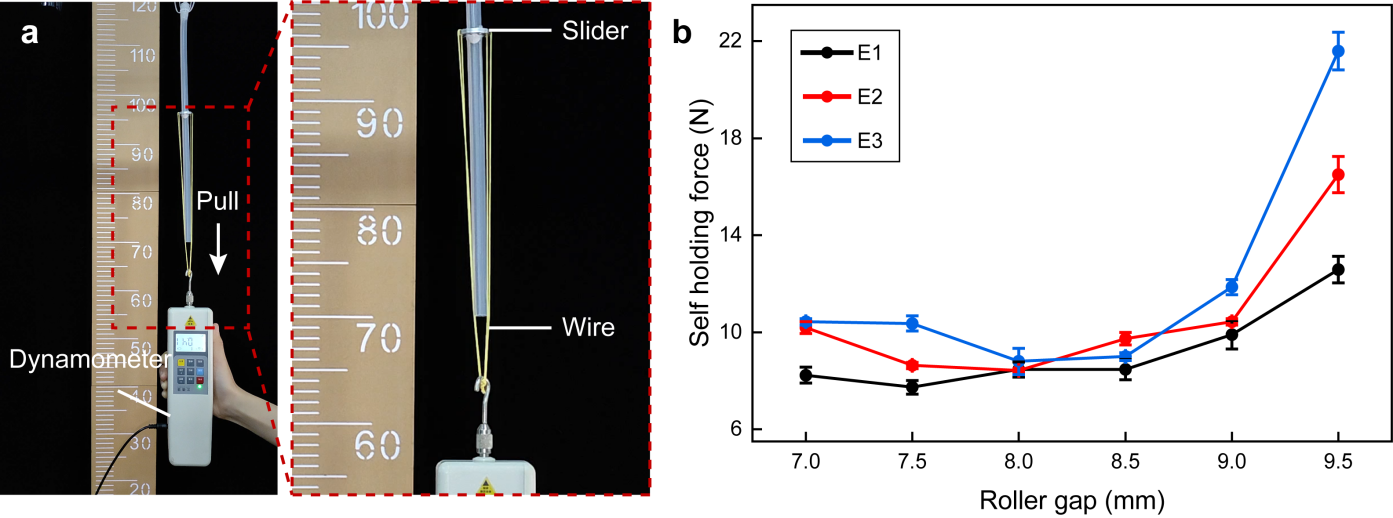


**Figure S10 | Self-holding measurement under different PFLA designs.** **a,** Experimental setup. The guide tube was hung vertically, and the slider was connected to a dynamometer directly below by an inextensible wire. The centers of the slider and dynamometer were vertically aligned in a straight line. A downward force was manually applied to the slider by the dynamometer and gradually increased until the movable system slid relative to the tube. At this point, the dynamometer value represented the self-holding force. When sliding occurred, the value displayed on the dynamometer represented the self-holding force of this design. **b,** The self-holding force was measured for roller gaps ranging from 7.0 to 9.5 mm. For guide tubes with three different elastic moduli, there exists a roller gap value that minimizes the self-holding force. As the elastic modulus increases, the roller gap value minimizing the self-holding force also increases. Specifically, for PFLAs using tubes with elastic moduli E1, E2, and E3, the roller gap values minimizing the self-holding force are 7.5 mm, 8.0 mm, and 8.5 mm, respectively. This is because the influence of the guide tube wrinkle effect becomes more pronounced with an increase in elastic modulus. Additionally, there is a slight increase in the overall self-holding force as the elastic modulus increases. However, the influence of the roller gap on self-holding force is more significant than the elastic modulus. The points are averages of three trials, and error bars show ±1 SD.


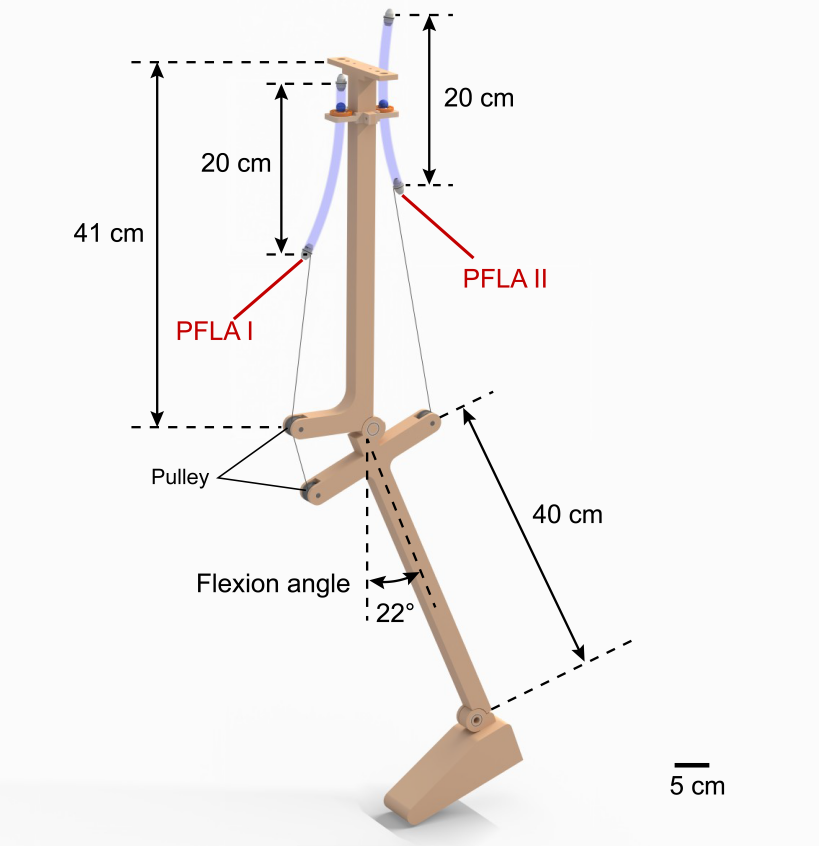


**Figure S11 | Design parameters of the wearable device and the prosthesis mimicking a human knee joint.** The joint model (241 g) imitated a female leg with a height of 160 cm. It was primarily made of photosensitive resin materials using a stereo lithography apparatus (SLA). Articulating joints were formed by connecting multiple bone segments using bearings. The joint model achieved a maximum knee flexion angle of 22°. Pulleys were installed at the points where the wire contacted the bone to reduce friction during wire movement.


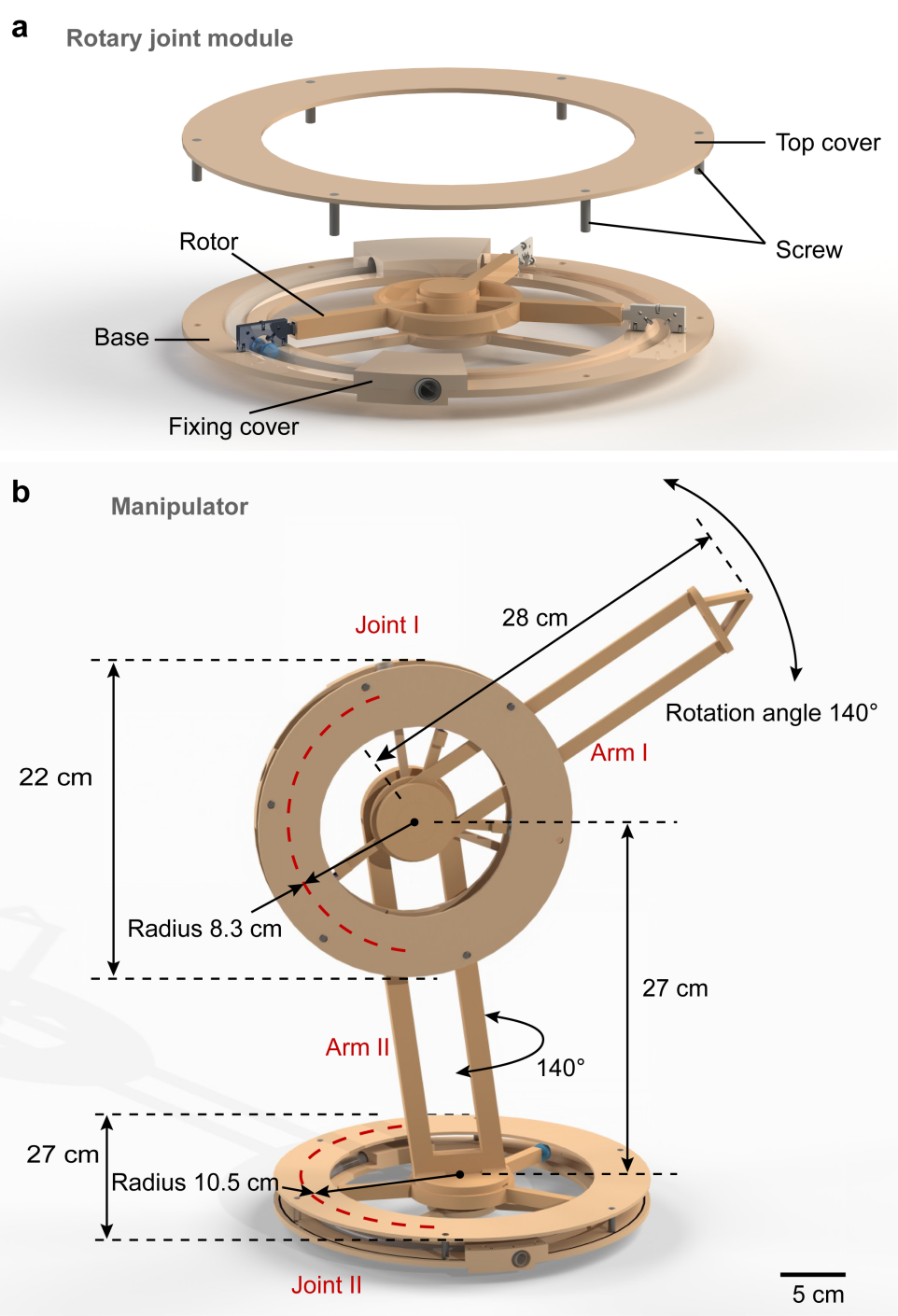


**Figure S12 | Design of the rotary joint module and the manipulator.** **a,** Design of the rotary joint module. The upper cover and base were fastened together using screws. **b,** The manipulator includes two joints and two arm rods. Joint I weighs 236 g and has a tube path with a radius of curvature of 8.3 cm, while joint II, weighing 332 g, features a tube path with a radius of curvature of 10.5 cm. The main components of the manipulator are fabricated from photosensitive resin materials using a stereo lithography apparatus (SLA).


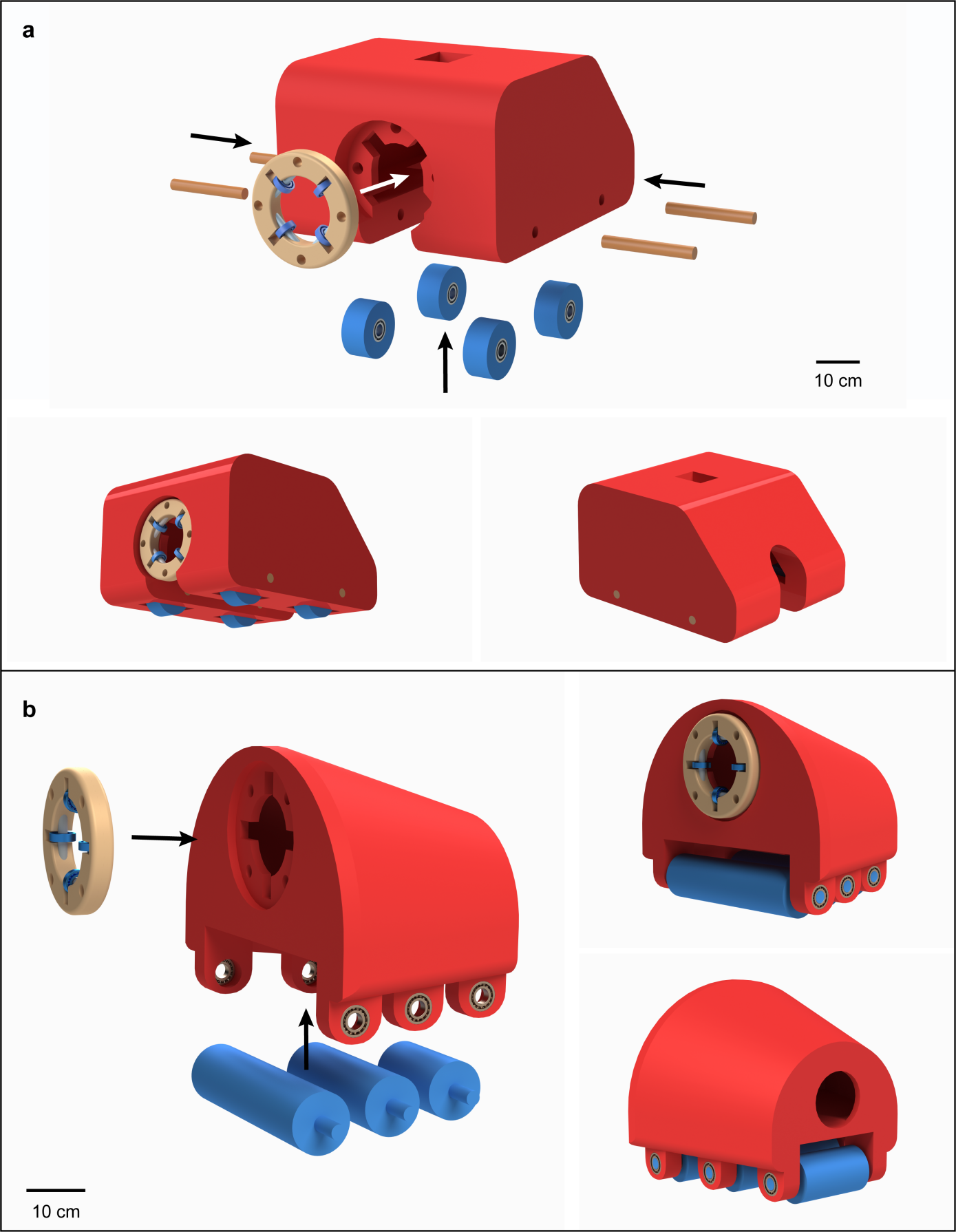


**Figure S13 | Two types of rail vehicles used in experiments. a,** The design employed in patrol firefighting. **b,** The design utilized in various environmental tasks. Both rail vehicles used H4 slider.

**Figure S14 | Cyclic test of the PFLA.** The piston reciprocated within a 1-meter-long guide tube, with each single trip counted as one cycle. The average speed per cycle was measured to assess wear on both the piston and the guide tube. Initially, we tested a piston with a 9 mm diameter (Piston 1) and observed that during the first 300 cycles, its speed fluctuated around 2.3 mm/s. As the cycles increased, the speed gradually rose, showing a 25% increase after 500 cycles. Measurements showed that the outer diameter of Piston 1 had reduced to 8.96 mm, a decrease of 0.04 mm. To determine whether the guide tube experienced wear, we tested a new 9 mm diameter piston (Piston 2) and observed a similar 25% speed increase after 500 cycles. This consistency suggests that the guide tube remains reliable after 1000 cycles. Additionally, it is advantageous that the piston can be easily replaced compared to the guide tube, thereby ensuring the continuous functionality of the system.

**Figure S15 | Comparison of response times for different tube lengths.** We tested the movable system with tube lengths of 1 m and 20 m, while varying the air pressure to observe the effects on response time. A displacement sensor was used to monitor the system's movement, with response time defined as the time required to reach 80% of the steady-state speed, and data averaged over three trials. The results show that as the tube length increases, the response time also increases. Conversely, higher air pressure reduces the response time. At pressures of 100, 150, and 200 kPa, the response times for the 1 m tube were 0.11, 0.1, and 0.07 s, respectively. For the 20 m tube, the response times were 0.7, 0.53, and 0.43 s, reflecting a 4 to 6-fold increase. However, all response times remained under 1 s. Due to the small inner diameter, only about 0.005 L of compressed air is needed per meter of tube during operation. Therefore, for general or short driving distances, the response time remains minimal. Although the response time increases significantly with longer tube lengths, we believe that the trade-off between driving distance and response time is acceptable.

**Figure S16 | Influence of pressurization rate on PFLA performance.** For the rapid pressurization test, we directly set the PFLA's input pressure to 160 kPa. For the slow pressurization test, we started at 90 kPa and incrementally increased the pressure by 10 kPa every 5 s until reaching 160 kPa. In both scenarios, we calculated the average speed at 5-second intervals and plotted the resulting speed variation curves. The results indicate that the PFLA's speed ultimately converges under both rapid and slow pressurization methods.**
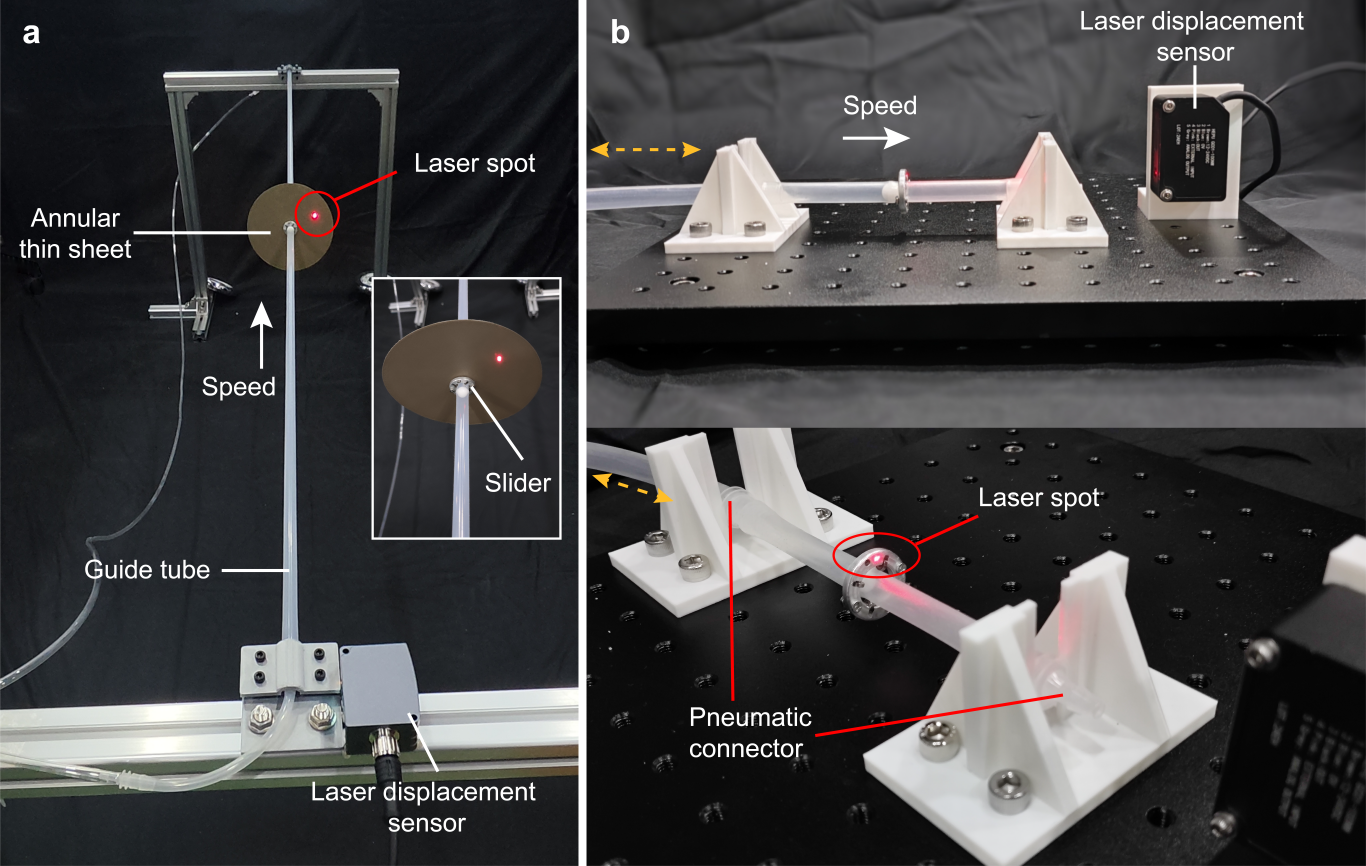
Figure S17 | PFLA speed monitoring setup. a,** Long-diatance speed monitoring setup. The test was conducted using a BOJKE (BLF-500NM-485) displacement sensor with a 10 Hz sampling frequency. Aluminum profiles were used to build two stands that held the ends of the guide tube, keeping it suspended. A 3D-printed annular thin sheet, 0.2 mm thick with an outer diameter of 15 mm, was mounted on the slider to provide a target for the sensor's laser. The sheet weighs 2.7 g, which has a negligible effect on PFLA movement. **b,** Response monitoring. This test was conducted using a HEPU (GC01-100NM) laser displacement sensor with a 100 Hz sampling frequency. The monitoring section of the guide tube were secured with 3D-printed components through pneumatic connectors. The laser of the sensor was directly aimed at the slider. By adjusting the length of the guide tube (indicated by the orange dashed lines), response monitoring at different distances was achieved.

**Figure S18 | PFLA's speed response under input pressure. a,** Speed response of the PFLA under square wave signal input. During the test, the input pressure was set to 110 kPa. After 55 s of pressurization, the air supply was removed and then re-applied after a 5-second interval. The results indicate that, at 110 kPa, the PFLA's average speed was 10.37 mm/s, fluctuating within the range of 10.05 to 10.82 mm/s, demonstrating high repeatability in control accuracy. **b,** Speed response of the PFLA under step signal input. The input pressure was set to 110 kPa. The results show that the response time of the PFLA (reaching 80% of the steady-state speed) was 0.12 s.

**Supplementary Table**

| Parameter description | H1 | H2 | H3 | H4 | H5 | H6 |
| --- | --- | --- | --- | --- | --- | --- |
| Roller gap (mm) | 7.0 | 7.5 | 8.0 | 8.5 | 9.0 | 9.5 |
| Maximum size (mm) | 21 | 22 | 22 | 22 | 22 | 23 |
| Weight (g) | 2.9 | 3.1 | 3.0 | 3.0 | 3.0 | 3.3 |
| Slider’s thickness (mm) | 3.5 | 3.5 | 3.5 | 3.5 | 3.5 | 3.5 |

**Table S1 | Specifications for six sliders used in experiments.** The term “Maximum size” refers to the outer diameter of the slider. The weight of the movable system is calculated as the sum of the weights of the slider and piston. The thickness of the slider is the combined thickness of two aluminium alloy sheets. The weight of the piston is 0.8 g.

**Description of Supplementary Movies**

**Movie S1. Actuation based on PPW principle.** This video demonstrates the actuation of the proposed PFLA based on the PPW principle. When input pressure is applied, the sealed chamber expands slightly, causing the piston to move forward under pneumatic pressure. This movement results in continuous deformation of the guide tube wall, which in turn pushes the slider's rollers to roll. To facilitate observation of the changes in the slider's position and the rotation of the rollers, we mark black lines on both the guide tube and the rollers using a marker.

**Movie S2. Expansion of sealed chamber under different air pressures.** This video shows the expansion of the sealed chamber at various input pressures at close range. The slider is fixed, and the left side serves as the air pressure input end. A ruler is used as a reference for measuring the expansion of the sealed chamber. When there is no air pressure input, the piston is in contact with the guide tube wall, forming a contact area with a certain width. At an input pressure of 50 kPa, the sealed chamber expands slightly, causing the contact area to narrow. At 100 kPa input pressure, the contact area further narrows. Finally, at 200 kPa input pressure, the contact area disappears completely, and a gap forms between the piston and the tube wall.

**Movie S3. Stability and anti-interference ability of the PFLA.** This video showcases the self-holding ability of the PFLA without input air pressure, demonstrated by hitting the movable system with a ping-pong ball and a 200 g weight. In part II, the PFLA's ability to operate under harsh conditions is further demonstrated through liquid spraying and violent shaking.

**Movie S4. Speed of the movable system under various input pressures.** This video demonstrates the speeds of the movable system under various input pressures. The speed of the movable system varies significantly within the pressure range of 90 to 200 kPa, indicating that the PFLA can achieve a large speed range with lower working air pressure.

**Movie S5. Visualization of air-release by FEM.** This video effectively visualizes the release of air from the sealed chamber using finite element method (FEM) simulations in COMSOL.

**Movie S6. Wearable device for leg assistance in humans.** This video demonstrates the potential of the proposed PFLA as a lightweight human limb assistive device. The assistive device, composed of two PFLAs, weighs 27 g. Part I showcases the completion of flexion-extension actions by the knee joint of the prosthesis with the assistance of the device. In part II, the device aids the prosthesis in ball-kicking.

**Movie S7. Needle threading and trajectory-tracking by the manipulator.** This video showcases the PFLA's precision driving characteristics and its potential applications in rigid systems. Part I demonstrates the manipulator's needle threading operation, guiding the wire through holes with diameters of 5 mm, 3 mm, and 2 mm, respectively. In part II, the laser device at the manipulator's end traces the letters 'S', 'H', and 'U' onto the board by adjusting the pressure input of the PFLAs. Through this actuation method, the manipulator achieves lightweight and energy-saving operation by externalizing power components.

**Movie S8. Versatile applications in challenging environments.** This video showcases various applications of the PFLA. Part I demonstrates the use of PFLA to detect the pipe with an inner diameter of 30 mm. The soft guide tube of the PFLA navigates the curved pipeline through passive deformation under external force, successfully detecting three preset fault points. In part II, the PFLA patrols along a predefined path and extinguishes the fire. Part III showcases the PFLA's application in different environmental conditions, including water, steps, and grassy areas

**Supplementary References**

[1] Usevitch, N. S. et al. An untethered isoperimetric soft robot. *Sci. Robot.* **5,** eaaz0492 (2020).

[2] Wakana, K., Namari, H., Konyo, M. & Tadokoro, S. Pneumatic flexible hollow shaft actuator with high speed and long stroke motion, *2013 IEEE International Conference on Robotics and Automation (ICRA)* (IEEE, 2013), pp. 357-363.
